# Habitat-use influences severe disease-mediated population declines in two of the most common garden bird species in Great Britain

**DOI:** 10.1038/s41598-022-18880-8

**Published:** 2022-09-05

**Authors:** Hugh J. Hanmer, Andrew A. Cunningham, Shinto K. John, Shaheed K. Magregor, Robert A. Robinson, Katharina Seilern-Moy, Gavin M. Siriwardena, Becki Lawson

**Affiliations:** 1 British Trust for Ornithology, The Nunnery, Thetford, Norfolk, IP24 2PU UK; 2 Institute of Zoology, Zoological Society of London, Regent’s Park, London, NW1 4RY UK

**Keywords:** Ecology, Conservation biology, Population dynamics, Urban ecology, Ecological epidemiology

## Abstract

The influence of supplementary feeding of wildlife on disease transmission and its consequent impacts on population dynamics are underappreciated. In Great Britain, supplementary feeding is hypothesised to have enabled the spread of the protozoan parasite, *Trichomonas gallinae*, from columbids to finches, leading to epidemic finch trichomonosis and a rapid population decline of greenfinch (*Chloris chloris*). More recently, chaffinch (*Fringilla coelebs*), has also declined markedly from the second to fifth commonest bird in Britain. Using citizen science data, we show that both declines were driven primarily by reduced adult survival, with the greatest reductions occurring in peri-domestic habitats, where supplementary food provision is common. Post-mortem examinations showed a proportional increase in chaffinch trichomonosis cases, near-contemporaneous with its population decline. Like greenfinches, chaffinches often use supplementary food, but are less associated with human habitation. Our results support the hypothesis that supplementary feeding can increase parasite transmission frequency within and between common species. However, the dynamics behind resultant population change can vary markedly, highlighting the need for integrating disease surveillance with demographic monitoring. Other species susceptible to *T. gallinae* infection may also be at risk. Supplementary feeding guidelines for wildlife should include disease mitigation strategies to ensure that benefits to target species outweigh risks.

## Introduction

The status of common bird populations is both an accepted indicator of wider ecosystem health^1^ and a direct influence on human wellbeing via interactions with nature^2^. Conservation attention most often focuses on rare and range-restricted species. Nevertheless, there are examples of rapid decline in previously widespread and abundant wild bird species at a scale that prompts conservation concern. Infectious disease can be a key driver of population dynamics in free-living wildlife, typically via modifications of host survival and/or reproductive success^3^ and there is increasing evidence of the emergence of novel diseases in, and their transmission between, wildlife species^4,5^. While most concern has been over zoonotic transmission^6,7^, infectious diseases are also of importance for wildlife conservation ^5,8,9^. For example, disease-mediated increases in mortality rates have been linked to population declines across multiple taxa, including birds^10–12^, mammals^13–15^ and amphibians^16^.

Increasing urbanisation can alter disease dynamics in wildlife^17–19^, and supplementary feeding, which is carried out by millions of households in the UK, has been shown to reshape bird communities^20–22^. Large multi-species congregations of birds can occur at supplementary feeding stations, repeatedly over many days, increasing the risk of intra- and inter-specific pathogen transmission beyond that likely to occur naturally^23,24^. Supplementary feeding is a tool with numerous conservation and management applications beyond the garden context (e.g.^25,26^), so an understanding of effects on disease occurrence and how this modulates the intended demographic impacts of feeding is critical for the design of guidance and policy.

An example of a recently emerged disease in wild birds is finch trichomonosis, caused by a clonal strain of the protozoan parasite *Trichomonas gallinae* that was initially detected in 2005 in Great Britain (GB)^27,28^. The parasite is predominantly transmitted between wild birds via fresh saliva, both directly through conspecific feeding, for example during courtship or rearing young, and indirectly via consumption of contaminated food or water^29^. Emergence of the clonal strain in GB is hypothesised to have occurred as a result of spillover from sympatric columbids, which are considered the primary host of *T. gallinae*, at supplementary feeding sites in peri-domestic habitats where passerines and columbids feed together^27^.

Finch trichomonosis causes upper alimentary tract lesions that interfere with the ability to swallow, leading to regurgitation, starvation and mortality^29^. Subsequent to the emergence of this disease, lethal trichomonosis has been detected most frequently in greenfinch *Chloris chloris* and chaffinch *Fringilla coelebs*, but also in a range of other passerine species in GB, some of which are of conservation concern (e.g. house sparrow *Passer domesticus*, bullfinch *Pyrrhula pyrrhula;* Robinson et al*.*^28^). While there appears to be sustained transmission within greenfinch and chaffinch populations, there is no evidence to date of sustained *T. gallinae* transmission or similarly large-scale trichomonosis mortality in other GB garden or farmland breeding passerines^30,31^.

**Figure 1:**
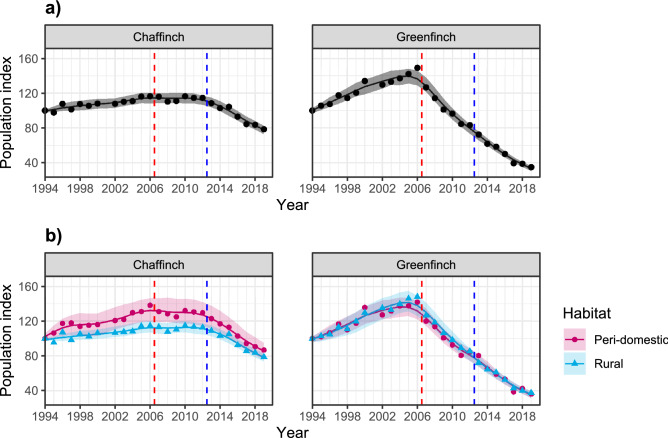
Breeding Bird Survey (BBS) population index trends for chaffinch and greenfinch for (**a**) GB overall (adapted from Woodward et al.^32^) and (**b**) GB survey sites identified as peri-domestic or rural (i.e. all other) habitat (BTO unpubl. data). The vertical, red, dashed line indicates the start of the greenfinch decline and chaffinch plateau period (early decline period), while the vertical, blue, dashed line indicates the start of the chaffinch decline period (late decline period). Plotted with 95% confidence intervals and indexed from 1994 (the first year of the BBS).

Large declines have been detected in greenfinch (from 2006) and chaffinch (from 2013) populations in GB (Fig. 1A). Whilst epidemiological evidence has previously supported trichomonosis as the primary driver of the greenfinch decline^23,31^, the cause of the chaffinch decline remains uncertain. Comparable population declines over the same timeline as the chaffinch and greenfinch declines have not been seen in other passerines in GB (Supplementary Fig. 1; Woodward et al.^32^). Thus, changes in climate or food availability that would be predicted to affect a range of species with shared habitat use or diet are considered unlikely explanations for the observed trends in the chaffinch population, and a disease-mediated decline—as has been shown for greenfinch—appears plausible. Disease dynamics that could produce such a pattern might have important implications in respect to understanding causes of change in wildlife populations.

Whilst direct transmission of *T. gallinae* may occur in these gregarious species across habitat types, we hypothesise that direct and indirect intra- and inter-specific contact rates are increased in the vicinity of supplementary feeding stations leading to more opportunities for transmission of this parasite. Female and juvenile birds often occur lower in the dominance hierarchy, so may be out-competed at feeders^33,34^, leading to greater parasite transmission among adult males, although not all studies have found this dominance hierarchy effect at feeders^35^. Alternatively, juveniles/females may be at a greater risk of mortality due to their competitive disadvantage, so patterns of trichomonosis occurrence and survival by sex and age could provide information about disease dynamics. We predict that there will be differences in survival trends between greenfinch and chaffinch and between species within peri-domestic and rural habitats, with consequent differences in population trends.

Here, we examine evidence from the monitoring of chaffinch and greenfinch abundance and demography at a national scale, together with post-mortem examination data, to (1) assess whether the comparative occurrence of trichomonosis in greenfinch and chaffinch has changed since the initial disease emergence and onset of epizootic mortality, especially in respect to the latter’s recent national decline, and whether the samples of affected birds are biased by age and/or sex; (2) quantify habitat-specific determinants of survival that may reflect differential disease impacts in peri-domestic versus rural habitats; (3) assess the overall contributions of different demographic rates to observed population change, given that impacts of finch trichomonosis are expected primarily to affect survival rather than breeding success. We use the results to infer the disease and population dynamics that underlie observed changes in abundance and their implications for wildlife populations more generally.

## Methods

### Species ecology and population context

The (common) chaffinch (*Fringilla coelebs*) and (European) greenfinch (*Chloris chloris*) are members of the finch family (Fringillidae) and are widespread in Great Britain and Europe. Despite the sustained declines in both species, chaffinch and greenfinch remain among Great Britain’s commonest breeding birds, with estimated breeding populations in 2016 of 4,800,000 and 760,000 pairs, respectively^36^ (down from 5,800,000 to 1,700,000 pairs in 2009, respectively^37^). The UK breeding populations of both species are largely resident, although chaffinch winter populations increase by ~ 30–50% with migrants from elsewhere in Northern Europe, while international movements to/from GB are rare for greenfinch^38–40^. Although they have similar diets and brood sizes, their life histories differ, with chaffinch typically being single-brooded, whereas greenfinch tends to make two breeding attempts annually. On average, chaffinch has higher adult and juvenile survival rates^41^. While both species occur across a wide range of habitats, greenfinch is more strongly associated with human habitation, whereas a greater proportion of the chaffinch population occurs in farmland^42,43^. Both species are highly gregarious and display strong flocking behaviour outside the breeding season in both single and multispecies flocks, including mixing with each other^44^.

Prior to 2006, population trends for both species had undergone long term increases, possibly linked to supplementary food provision^22^ and/or milder winters^45^. Since then, three key periods of consistent population trend are apparent (Fig. 1a): (1) the period before the onset of the epizootic of finch trichomonosis (2000–2006); (2) the period between the start of the greenfinch decline and the start of the chaffinch decline during which the latter’s population trend plateaus (2007–2012); (3) the period of both chaffinch and greenfinch decline (2013–2019). We hereafter define these periods as (1) pre-decline, (2) early decline and (3) late decline; complete digitised demographic data for estimating survival and productivity are not available before 2000.

The population trends for the two species can also be considered over two major habitat type groupings in Great Britain (following Crick^46^): peri-domestic, i.e. domestic gardens around human habitation along the rural–urban gradient where provision of supplementary food for wild birds is common practice^47^, and rural, grouping together all other terrestrial habitat types that are not dominated by human habitation and where supplementary feeding, at least of the type practised in gardens, is rarer. Both species occur commonly in the two habitat types^44,48^. Trends for greenfinch were near-identical in peri-domestic and rural habitats, whereas the trend for chaffinch in peri-domestic habitats increased more steeply than in rural habitats in the pre-decline period (increases of 19% and 8% 2000–2006), plateauing at a higher relative level in the early decline period (Fig. 1b). Furthermore, in the late decline period, chaffinch showed a slightly greater decline in peri-domestic versus rural sites (declines of 33% vs. 30%).

### Post-mortem examination data

Scanning disease surveillance of passerines was conducted across Great Britain from 2005, when the emergence of finch trichomonosis was first detected^28^, to 2019, inclusive, through two, consecutive, national citizen science programmes. Members of the public reported sightings of sick or dead passerines, typically found in or near gardens, to the Garden Bird Health *initiative* (GBH*i*), 2005–2013, and subsequently to the Garden Wildlife Health Project, 2013–2019, which incorporated the GBH*i*. When possible, well-preserved carcasses of all species were submitted for post-mortem examination (PME). All wild birds examined in this study were found dead or euthanised for welfare reasons. No permit, or further approval, was required to obtain, hold or use these samples. Origin locations were recorded to 1 km square Ordnance Survey grid reference (‘sites’). For species and age confirmation, digital photographs were taken by the responsible veterinarian of a ventral view of the body, an extended dorsal wing and the tail plumage, as part of the PME protocol.

To establish cause of death, submitted carcasses underwent a systematic examination of external and internal body systems, supported by microbiological, parasitological, histological and molecular diagnostic testing as appropriate, based on macroscopic findings^28,49^. A case definition was employed for finch trichomonosis, following Robinson et al*.*^28^ (See Supplementary Material, Appendix 1 for further details). To facilitate analyses, cause of death was categorised as: trichomonosis (confirmed or suspected), other infectious disease (e.g. cnemidocoptosis, *Escherichia albertii* infection, salmonellosis), trauma/predation, or undetermined.

To examine changes in the occurrence of finch trichomonosis over time, two different approaches were used. First, for each year of the study, the annual proportion of passerine carcasses examined which had trichomonosis were calculated separately for each of chaffinch, greenfinch or other passerines. Second, as multiple trichomonosis cases could be submitted from the same site, the annual proportion of sites where at least one trichomonosis case in each category of bird was submitted was calculated for each year, as was the annual proportion of sites that submitted at least one trichomonosis-positive carcass of any species of passerine. To align with the demographic analyses (see later), the annual period (termed “recovery year”) was defined as July–June.

This PME dataset relies on submissions by citizen scientists and represents a non-random (or convenience) sample, and, as a garden-based study, will be strongly biased towards peri-domestic sites and regions with human habitation (sites are mapped in Supplementary Fig. 2). Observations of sick and dead wild birds may be more likely in gardens where birds are fed, and closely observed, than in other gardens. Whilst reports of all perceived causes of wild bird mortality, including trauma and predation, are solicited and investigated as part of this disease surveillance programme, motivation to report signs of ill health may be increased when multiple mortality of wild birds, consistent with infectious disease outbreaks such as those due to trichomonosis, occurs, resulting in further skew. However, no differential selection of cases for submission was practised by species (i.e. chaffinch versus greenfinch or other passerine) or age during the study; therefore the data are considered to be informative on the changes in trichomonosis occurrence amongst passerine species.

The digital photographs of chaffinches and greenfinches were reviewed by a licensed bird ringer (H.J.H.) who assigned age (either juvenile, in their first year or adult older birds) based on month of death, and sex^50,51^, to each carcass. Carcasses with age characteristics that could not be reliably assessed due to image clarity and/or damage/degradation (15% of chaffinch and 6% of greenfinch photographed carcasses) were excluded from analysis. For greenfinch and chaffinch, we used within-species chi-squared tests to examine whether trichomonosis was found disproportionately in either age-class or sex.

### Habitat-specific survival

To investigate whether habitat-specific population trends were linked to variation in survival rate, we undertook habitat-specific survival analyses using reports of ringed birds found dead^52^, classified by primary capture habitat into the same peri-domestic versus rural site categories as the population trend data. Fledged birds are aged at the time of ringing, based on plumage characteristics, as first-winter or adult birds. We considered only birds ringed between April and September (inclusive) as these individuals generally form a discrete British population, with little interchange with other populations in continental Europe^38^. All birds were captured and ringed by trained bird ringers licensed by the BTO (British Trust for Ornithology) following all relevant guidelines and regulations.

Individual age- and habitat-specific survival probability was estimated using standard multinomial models of mark-recovery data^53^. Within the same model, annual survival (*φ*_*t*_) was estimated for first-winter birds, from fledging to 30th June the following calendar year (${\varphi }_{fw,h,t}$) and, for adults, from 1st July to the following 30th June ${(\varphi }_{ad,h,t})$ in each of the two habitats (*h*). Birds of uncertain age at the time of ringing were excluded. Age-specific survival was modelled using habitat- and period- specific means ${\mu }_{h,p}$ with annual residuals ${\varepsilon }_{h}\sim N(0, {{\sigma }^{2}}_{h,p})$. Furthermore, to assess whether survival in either age-class or habitat had changed between the identified periods of interest, the mean static survival probability with associated annual residuals ε[t] was modelled separately for each of the three identified periods (2000–2005, pre-decline; 2006–2012, early decline; 2013–2019, late decline). The difference between the mean survival for these three block periods was then calculated. Note that, as survival for the final year of data (birds ringed in 2019) cannot be fully assessed due to the truncated potential reporting period, this was not included in the mean periods.

The reported number of individuals ringed in a given year’s cohort that are recovered in year $t$ is a product of the age-specific annual survival probabilities $(\varphi )$ from the period of ringing up to $t-1$, the probability of mortality in year $t (1-{\varphi }_{t})$, and the probability that, having died, a ringed individual is found and reported (${p}_{t}$). This reporting probability was also modelled as an age and habitat-specific mean ${\mu }_{a,h}$ over the entire study period, with annual residuals ${\varepsilon }_{t}\sim N(0, {{\sigma }^{2}}_{a,h})$. Although there may be a bias towards reporting from gardens and other peri-domestic settings, this is likely to be similar amongst the three periods.

It was assumed, for simplicity, that birds ringed in one habitat type are representative of birds using that habitat regularly, based on the typically short distances moved by birds between ringing and the recovery sites in the present dataset (median = 0 km, inter-quartile range [IQR] = 0–2 and median = 2 km, IQR = 0–5 for chaffinch and greenfinch, respectively). However, if this assumption is incorrect and there is more interchange between habitats, which is possible based on within GB-movements, particularly for greenfinch^38^, differences in survival would be expected to be diluted by confounding influences of multiple habitats, thus making our analyses conservative.

The model was fitted in a Bayesian paradigm via Program R^54^ using JAGS^55^ via the R2jags package (version 0.6–1; Su & Yajima^56^); all code including priors is provided in Supplementary Material, Appendix 2. The sampling process was run for 100,000 iterations with three independent chains run in parallel on separate cores to increase speed, a burn-in period of 50,000 iterations and a thinning value of 10 (i.e. 15,000 iterations were sampled). Model convergence across the chains was assessed using the Brooks-Gelman–Rubin statistic $\widehat{R}$, with all $\widehat{R}$ < 1.05. We also inspected output trace plots to confirm effective burn-in and chain mixing across the full parameter space. We report the posterior means and the Bayesian 95% credible intervals with inter-quartile (50%) intervals included in some plots.

### Demographic contributions towards population changes

To assess the relative importance of survival compared to productivity in explaining population change, we constructed an integrated population model (IPM) for each of the two species. Insufficient productivity and movement data were available to build habitat-specific IPMs, so these IPMs were for GB overall, regardless of habitat. We combined data from three, national, long-term citizen science monitoring programmes operated by the BTO between 2000 and 2019 into an integrated population model, largely following Robinson et al.^57^: (1) population trend data from the BTO/RSPB/JNCC Breeding Bird Survey (BBS)^58,59^; (2) mark-recovery survival data from the BTO bird ringing scheme for adult and first-winter birds pooled across all habitats; and (3) nest productivity and survival data from the BTO Nest Record Scheme (NRS; Crick et al.^60^). This IPM analysis assumes that these data sources are independent of each other, which in reality is unlikely to be strictly true; however, previous studies have suggested that this is unlikely to bias the results substantially^61–63^. All nest monitoring was carried out on behalf of the BTO following the NRS Code of Conduct and does not require specific licensing for these species. Full details of the population model used and the IPM fitting including JAGS code are included in the Supplementary Material, Appendix 3; for further details of the population model and associated datasets used in this study, see Robinson et al.^57^.

Life table response experiments (LTREs) were used to analyse the overall contribution of the variation in individual demographic parameters to the variation in population growth λ^64–66^ in the IPMs using the following equation:

1 $$Contribution_{{\theta_{i} }}^{{var\left( {\lambda_{t} } \right)}} \approx \mathop \sum \limits_{j} cov\left( {\theta_{i,t} ,\theta_{j,t} } \right)\frac{{\partial \lambda_{t} }}{{\partial \theta_{i,t} }}\left. {\frac{{\partial \lambda_{t} }}{{\partial \theta_{j,t} }}} \right|_{{\overline{\theta }}}$$

where $\partial {\lambda }_{t}/\partial {\theta }_{i,t}$ is the sensitivity of the population growth rate with respect to ${\theta }_{i}$, and $i$ and $j$ are indices for demographic rates and population structure, respectively. From this, the contributions to variation in λ were estimated as the sums of the products of the pair-wise covariances between the demographic parameters and the sensitivity matrices. Sensitivities were calculated via the popbio R package (version 2.7; Stubben & Milligan^67^). Results were calculated across all saved IPM model iterations comprising the posterior distributions for each model parameter. The results were then scaled to sum to 1 for each model iteration, for ease of comparison, and are summarized as means, followed by lower and upper limits of the 95% credible intervals.

**Figure 2:**
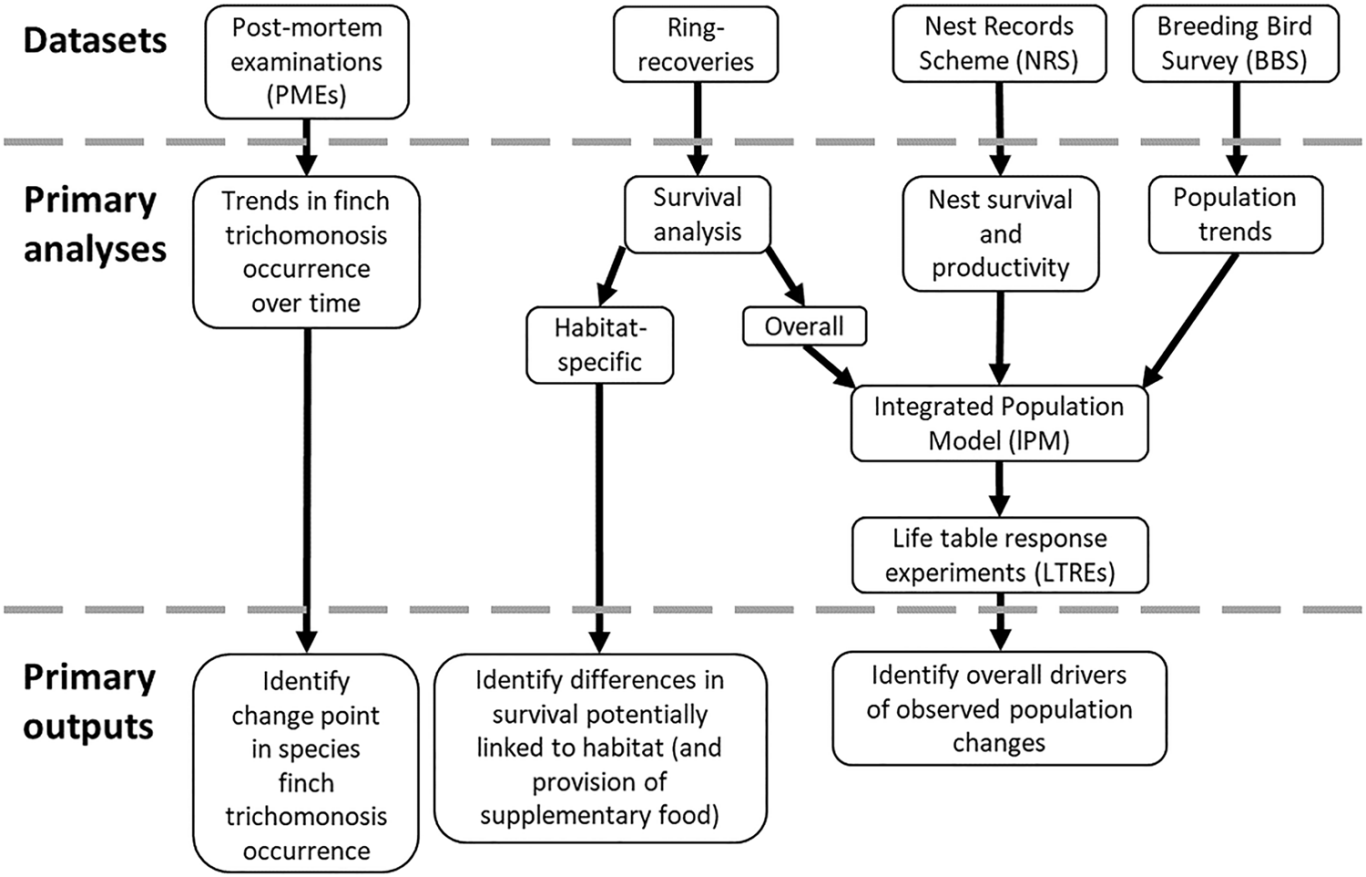
Schematic overview of the datasets and analyses utilised in this research, and their primary outputs.

A schematic overview of the datasets and analyses utilised in this research and their primary outputs is provided in Fig. 2.

## Results

### Post-mortem examination results

Post-mortem examinations were conducted on 2184 individuals of 49 passerine species found dead predominantly in garden habitats between July 2005 and June 2019: 30% were greenfinch and 18% were chaffinch (Supplementary Table 1). Cause of death was confirmed or suspected due to trichomonosis in 961 (44%) cases involving 14 species, with the majority of these being greenfinch and chaffinch (52% and 29% of trichomonosis diagnoses). The next three most affected species, goldfinch *Carduelis carduelis*, bullfinch and house sparrow were diagnosed with the condition much less frequently (7%, 4% and 2% of trichomonosis diagnoses, respectively).

The carcasses examined came from 1296 unique sites (1 km square grid references), with carcasses being submitted for PME from a median of 101 sites (IQR = 82–129) per recovery year. At least one trichomonosis case was identified from 54% of sites with a median of 52% per recovery year (IQR = 47–57%). Multiple carcasses were submitted for examination from around a quarter (28%) of sites within the same recovery year, although fewer sites (12%) submitted multiple birds with trichomonosis within the same recovery year. Within-recovery-year confirmation of trichomonosis in multiple species was recorded at 4% of sites and specifically involved both greenfinch and chaffinch at 2% of sites.

**Figure 3:**
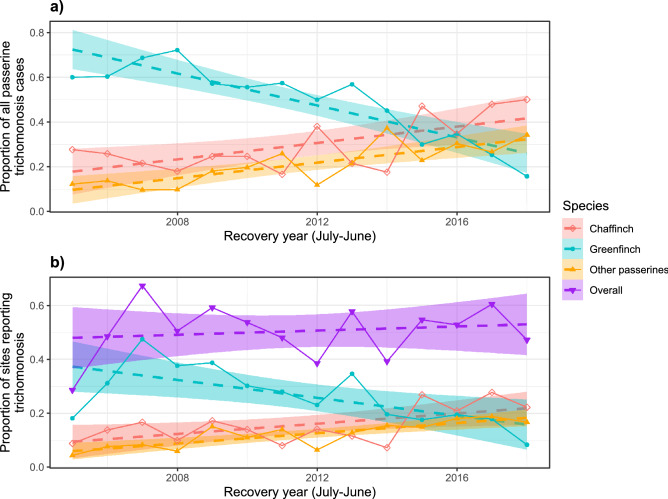
(**a**) Proportions of post-mortem examinations confirmed or suspected to be trichomonosis per recovery year for chaffinch, greenfinch and other passerine species; (**b**) proportion of all sites that submitted carcasses that included at least one passerine trichomonosis case overall and specifically for chaffinch, greenfinch and other passerine species per recovery year. The recovery year ran from July to June to match the period used for calculating survival estimates. Plotted with simple linear model lines of best fits with 95% confidence intervals.

The proportion of passerines diagnosed with trichomonosis accounted for by greenfinch fell over time, with a switch from greenfinch to chaffinch as the modal species occurring around 2014/15 to 2015/16 (Fig. 3a). Similarly, while the proportion of all sites submitting carcasses for PME that were found to have trichomonosis has remained broadly stable since 2006, the proportion of those sites from which greenfinches with trichomonosis were submitted fell over time while the proportion of sites with chaffinch and other passerine species trichomonosis cases rose, with the switch also happening around 2015 (Fig. 3b). Note that the proportion of sites reporting trichomonosis in 2005, the first year in the dataset, was lower, as this was the initial year of trichomonosis emergence, when it had a restricted regional distribution; national epizootic mortality was not observed until 2006^28^.

Of the examined carcasses, 64% of greenfinch and 77% of chaffinch were photographed, with age being determined for the majority (94% and 85%, respectively) of each species. For chaffinch, 65% of known-age birds were adult, compared to only 38% of greenfinch. With chaffinch, there was no evidence for significant differences between the age categories for different causes of death (χ^2^_3_ = 2.29, *p* = 0.51; Supplementary Fig. 3a). For greenfinch, however, a higher proportion of young birds were diagnosed with predation or other trauma than any other cause of death (χ^2^_3_ = 10.2, *p* = 0.017, Supplementary Fig. 3a). Marginally more males were submitted for PME than females in both species (56% male for chaffinch, 54% for greenfinch). A higher proportion of males than females was diagnosed with trichomonosis, but this sex ratio did not differ significantly from that in the other cause-of-death categories (χ^2^(3) = 3.25, *p* = 0.35 for chaffinch, χ^2^(3) = 1.36, *p* = 0.71 for greenfinch; Supplementary Fig. 3b).

### Habitat-specific survival trends

**Figure 4:**
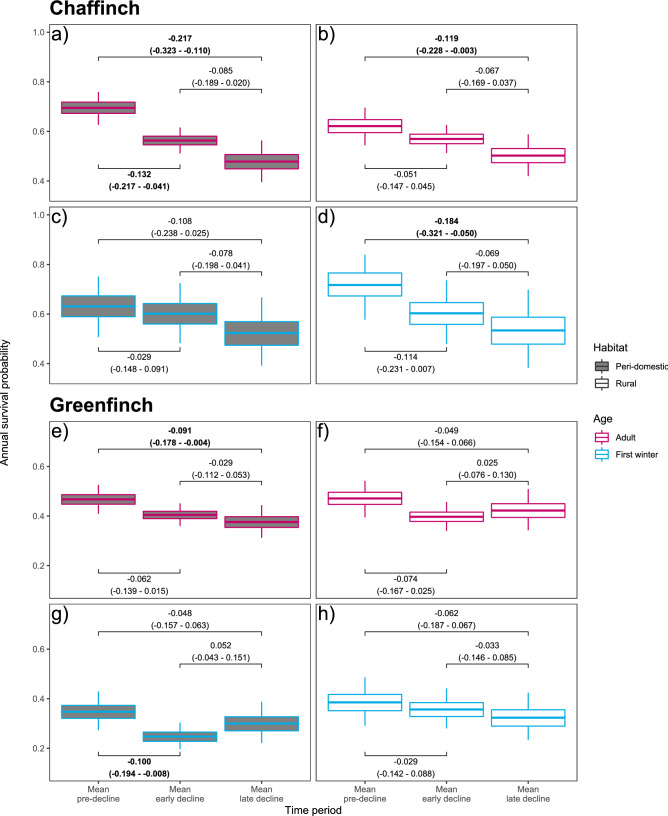
Mean habitat-specific annual survival estimates for (**a**–**d**) chaffinch and (**e**–**h**) greenfinch between 2000 and 2018, with mean survival blocks for the pre-decline (2000–2005), early decline (2006–2012) and late decline (2013–2018) periods, from the respective habitat-specific mark-recapture models. In the plots, bars show the mean, boxes the 50% and whiskers the 95% credible intervals (CI) calculated via posterior density intervals. In the labels the top lines show the mean change in mean survival between the relevant block periods and the second line is the 95% CI for the change in survival. Labels where the 95% CI did not overlap zero are displayed in bold. The recovery reporting probabilities were species, age- and habitat-specific.

Survival patterns differed between species, age-classes and habitats (Fig. 4; Supplementary Table 2). For chaffinch adults, mean survival decreased considerably in peri-domestic habitats during both the early and late decline periods compared to the pre-decline period (30% reduction overall), while the overall reduction was smaller in rural habitats (19% reduction; Figs. 4a,b). First-winter chaffinch survival, however, declined less in peri-domestic versus rural habitats (overall reductions of 17% and 26% respectively; Figs. 4c,d). Consequently, pre-decline period adult chaffinch survival was 11% higher in peri-domestic habitats whereas it was 4% lower in the late decline period compared to rural habitats (Figs. 4a,b), while rural first-winter survival was higher (13%) in the pre-decline period, with no evidence for a real difference in late decline period between the two habitats (mean first-winter survival was 2% higher in peri-domestic habitats; Figs. 4c,d). For greenfinch adults across both peri-domestic and rural habitats, and for greenfinch first-winter birds in peri-domestic habitats, mean survival reduced considerably during the early decline period compared to the pre-decline period (reductions of 15%, 15% and 29% respectively; Figs. 4e,g), with the decline being less marked in first-winter birds in rural habitats (8% reduction; Fig. 4h). Between early and late decline periods, both rural adult and peri-domestic first-winter greenfinch survival recovered slightly following the initial drop (increases of 5% and 20%). Survival rates continued to fall for peri-domestic site adult and rural site first-winter greenfinches (further reductions of 5% and 11% between early and late decline periods). Overall, greenfinch survival fell more at peri-domestic sites, compared to rural sites, for adults (mean reductions of 19% versus 11%), but the opposite was true for first-winter birds, with a larger reduction in survival at rural sites (mean reductions of 14% versus 18%).

Recovery reporting probabilities differed by species, age and habitat. For both species, reporting rates were higher in peri-domestic habitats. For chaffinch, dead, marked first-winter birds were reported more often than adults, whereas for greenfinch the converse pattern occurred (Supplementary Table 2).

### Demographic contributions towards population changes

**Figure 5:**
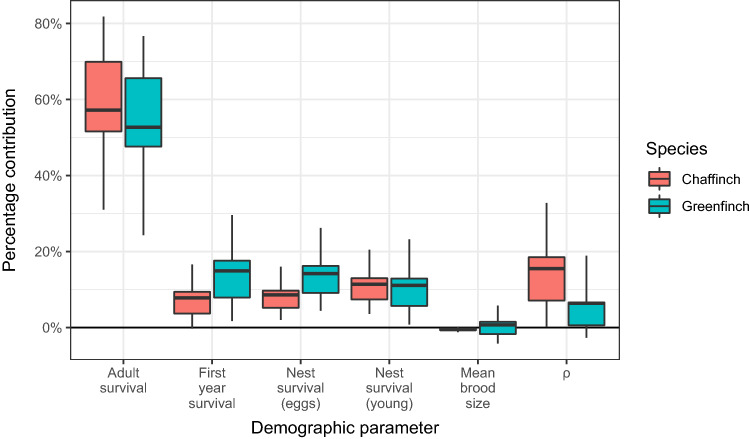
The overall percentage contribution of measured demographic parameters to population growth rate $(\lambda )$ for chaffinch and greenfinch in Great Britain between 2000 and 2019 calculated using Life Table Response Experiments. For an explanation of the boxplots see Fig. 4.

For both species, LTREs showed adult survival to be the single largest contributor to realised population change; measures associated with breeding success (nest survival for both eggs and young) contributed much less, as did survival over the first winter period (Fig. 5). Changes in brood size had a negligible effect on demographic change in both species. The combined contribution of $\rho$, the demographic parameters for these species that cannot be easily directly measured (number of breeding attempts per pair, post-fledging survival and breeding propensity) made a greater (but still small) contribution to population change for chaffinch than it did for greenfinch.

Plots of all estimated annual demographic rates can be found in Supplementary Fig. 4, with mean demographic rates included in Supplementary Table 2.

## Discussion

Our study indicates that large national population reductions (in the order of 2.5 million breeding pairs overall) of greenfinch and chaffinch, both widespread and common bird species, have been largely driven by lower adult survival associated with the emergence and epidemic spread of finch trichomonosis, although the timing of these declines differed. The population impact on greenfinch began around the initial outbreak of finch trichomonosis in 2006 and appears to have continued since then, whereas the impact on chaffinch populations has been more complex. Due to the magnitude of its population decline, greenfinch has become one of the few species in the UK to move directly from the lowest to the highest category of conservation concern, and the first one to be listed because of infectious disease^68^. In both species, adult survival fell to a greater extent in peri-domestic than in rural habitats following the onset of the finch trichomonosis epizootic.

Greenfinch and chaffinch both display strong flocking behaviour outside the breeding season, often forming flocks together (as well as with other species; Newton^44^). This is likely to have facilitated wider inter- and intra-specific transmission, especially at food sources, within and between different populations. The annual migration behaviour of chaffinch to and from GB is considered likely to have initiated the spread of the clonal strain of *T. gallinae* responsible for the finch trichomonosis epizootic through continental Europe^30,69^, indicating that migratory birds may be infected by *T. gallinae.* Infected migratory chaffinches also could have spread the disease amongst resident UK finch populations, both in peri-domestic and rural settings. Despite identification of the chaffinch as the probable primary vector of spread to the continent, there is little evidence to date for any significant impact of trichomonosis on wider European chaffinch populations, which are considered to be largely stable^70,71^. However, greenfinch regional trends appear more mixed with declines in the north and west of Europe, and increases in the south and east of the continent^70,71^. Even within the former area, greenfinch population trends following the emergence of finch trichomonosis have varied; for example, increasing or stable in the Netherlands^72,73^ but declining in Finland^69^. In part, these differences may reflect differing habitat preferences and behaviour. Chaffinches are more strongly associated with old growth forest in mainland Europe than they are in the UK^71^, which, combined with their stronger territorial breeding season behaviour, and consequent lower breeding population density^44^, may have helped to reduce parasite transmission rates in their populations. Conversely, greenfinches use a much wider range of habitats across Europe, with no strong regional differences in habitat preference^71^, which, combined with their more social breeding season behaviour and higher breeding population density^44^, may place them at greater risk of exposure once finch trichomonosis enters a population. The observed regional differences could also be linked to differing national attitudes towards supplementary feeding of wild birds across Europe. While large-scale supplementary bird feeding is carried out within at least 18 European countries, mostly in the north and west, the majority only do so during the winter, whereas, in the UK, year-round feeding has been advocated in recent decades^74^, with the supplementary feeding industry growing considerably in that time^22^. This may have increased the potential exposure of greenfinches, which have declined mostly in the regions of Europe where feeding is common^70,71^, to *T. gallinae*.

Linking the changes in survivorship and population trends directly to changes in finch trichomonosis prevalence remains challenging. However, using PME data as a proxy for the passerine species composition affected during the epizootic, we show that, from 2015, soon after its population started to decline, chaffinch became the species most frequently diagnosed as having died due to trichomonosis. The main initial effect of the epizootic appears to have been to suppress chaffinch population growth, following a long period of sustained increase, which, in turn, occurred as part of a wider shift in urban bird community structure^22^. The impact of the disease on chaffinch survival appears to have been most marked at peri-domestic sites, consistent with aggregations of individuals around supplementary food sources increasing transmission risk^23^.

As greenfinches have declined, chaffinches may have become more able to utilise food sources previously monopolised by greenfinches and so increased the rates of exposure to, and transmission of, *T. gallinae* amongst chaffinches at bird feeders. However, there is little evidence of an increase in relative garden use by chaffinches following the decline of greenfinches which might have been expected if this were true (BTO Garden BirdWatch; www.bto.org/gbw) although the data may currently lack sufficient resolution to detect these effects if they exist. In addition, the BBS trend for chaffinch numbers in peri-domestic habitats, where supplementary food is most likely to be encountered, has mirrored the rural BBS trend throughout.

The ratios of adults to first-winter birds and of males to females examined post mortem were similar overall for all causes of death considered within each species. This suggests that the adult age class has not been disproportionately affected by trichomonosis and indicates that the transmission of and morbidity from this disease are not strongly condition- or dominance-related. Likewise, beyond a slight bias towards males in most categories, as has been observed with some other infectious diseases of passerines^75–77^, no differential effect on sex by trichomonosis was found.

There are various possible explanations for the observed differential impact between chaffinch and greenfinch, at least immediately following the emergence of finch trichomonosis, some of which are complex. Greenfinches could plausibly be innately more susceptible to infectious diseases and other stressors, as might be indicated by some authors (e.g.^49,78^). Alternatively, or additionally, species differences in *T. gallinae* infection dynamics, such as differences in exposure rates, time from infection to infectiousness and intra-specific transmission rates, could influence the timing and degree of population impacts. The larger, more aggressive greenfinch, for example, tends to be dominant over chaffinch and most other species at bird feeders, while interacting aggressively with conspecifics^79,80^. This may increase the risk of contamination of, and exposure to, feeders with *T. gallinae*, in addition to direct conspecific transmission through salivary exchange. Greenfinch primarily feed their young via regurgitation^81^, an important route of *T. gallinae* transmission in columbids through crop milk^29^; in contrast, chaffinch mostly feed their young whole food items, which may reduce the risk of parasite transmission. We found little evidence of any change in breeding success playing an important role in driving the observed population change of greenfinch, therefore nestling mortality resulting from parasite transmission from adult to young is not supported as a major driver. In addition, during the breeding season, chaffinches tend to hold more distinct territories than greenfinches^44,81^, so even though the species is more numerous^36^, their probability of close contact and subsequent disease transmission is reduced during that part of the year. Another possible explanation for the different patterns seen in the population responses to trichomonosis include an increase over time in the susceptibility of chaffinch to *T. gallinae* infection, perhaps due to the development by the parasite of increased virulence. Studies, such as epidemiological modelling, infection challenge experiments and characterisation of *T. gallinae* virulence determinants, are required to explore these hypotheses further.

Regardless of why there were differences in the timing of population declines, it is noteworthy that abundances have continued to fall. Assuming density-dependent transmission and/or demographic compensation^82^, we would predict a swift stabilisation of population trends with reduced greenfinch and chaffinch numbers, as was found after the emergence and epizootic spread of the bacterial disease, mycoplasmosis, in passerines in North America^83,84^. However, if sympatric species act as parasite reservoirs, such as columbids^27,85^, infection may continue to occur, particularly at supplementary feeding sites where greater numbers and species complements repeatedly gather within a small area, fostering continued declines.

Notwithstanding the potential complexities outlined above, habitat appears to have played a clear role in the decline of chaffinch, if not greenfinch. The differential patterns of survival suggest that populations further away from human habitation may have supplemented peri-domestic site populations, initially stabilising chaffinch populations. However, a greater understanding of population interactions between habitats is required to test this hypothesis. Supplementary feeding is ubiquitous in peri-domestic sites in the UK^47,86^. Therefore, as feeding stations have previously been identified as a key point for both intra- and inter-species disease transmission, due to the high bird densities and large species complements that are attracted to them (e.g.^23^), facilitation of trichomonosis spread at feeding stations might explain both the maintenance of the population declines and the differences in habitat-specific adult survival.

Further to trichomonosis becoming widespread within the greenfinch and chaffinch populations in the UK, there is increased potential for spillover of *T. gallinae* to other susceptible species using supplementary feeding stations. Indeed, fatal trichomonosis has already been diagnosed in a range of passerines, albeit in relatively small numbers overall (Table S1), with cases increasing over time and sometimes occurring at sites with concurrent greenfinch or chaffinch mortality (B.L. & K.S.M. unpublished observations, Robinson et al*.*^28^). The inter-specific spillover of a disease from a common species into rarer species may be of concern, for example, a spill-over event of *T. gallinae* from greenfinches to white-winged snowfinches (*Montifringilla nivalis*) at a bird feeder in a Swiss mountain village resulted in multiple mortalities of this endemic, alpine species^87^.

The disease-mediated decline of two very common and widespread species highlights the importance of investigating the ecology of pathogens as a driver of wildlife population dynamics and the need for continuing health surveillance combined with mitigation research. It is particularly noteworthy that concurrent infections of different species in a community with the same parasite can lead to large population declines across different time periods. This suggests that there could be latent demographic impacts of trichomonosis that are not yet realised in other species, such as the red-listed hawfinch *Coccothraustes coccothraustes*, house sparrow, lesser redpoll *Acanthis cabaret* or yellowhammer *Emberiza citrinella*, from which fatalities due to the disease have also been identified (Supplementary Table 1).

This research relies on data collected through volunteer citizen science schemes, which have particular strengths and weaknesses in the present context^88–90^. BBS population trends are generated from an annual, structured, randomised, transect survey covering all terrestrial habitats, which should minimise spatial and temporal biases in the dataset^58,59^. The other schemes utilised here to a greater or lesser extent represent convenience samples, especially with regard to site and habitat selection, and so may produce results that are not truly representative of the status of these species and the impacts of trichomonosis across GB. However, spatial biases in reporting are likely to favour areas closer to human habitation, but this pattern is unlikely to have changed over time during the study period, so temporal biases are unlikely to have been introduced. Our results show the value of citizen science scheme data for multidisciplinary research, in addition to their vital importance for biodiversity monitoring worldwide, for which they often represent the only practical and cost-effective approach to data gathering (e.g.^60,91,92^). The finding and reporting of dead wild birds for PME or as ringing recoveries, especially on restricted access land such as private gardens, is only viable, at any scale, via citizen science schemes. Furthermore, far greater numbers of sites can be covered cost-effectively with volunteer participation than using paid surveyors, such that data quantity typically outweighs potential issues with quality or spatial bias^88,92,93^. In addition to the value for data and evidence provision, citizen science may benefit individual mental health and public interest in the natural world, which may in turn enhance conservation outcomes^94–96^.

The results of the current study put a spotlight on the use of supplementary feeding, both in gardens and in a conservation management context. While supplementary feeding has known population benefits for a range of bird species^22,25^, the possibility of negative and counteracting effects on species conservation and animal welfare needs to be considered along with the promotion and employment of appropriate mitigation measures^20,97,98^. This has implications for targeted supplementary feeding used in conservation projects, such as the provision of seed for farmland birds to augment depleted natural food sources^25,99^. The extent to which the details of feeding activity, such as summer versus winter feeding, or poor awareness, adoption, or efficacy of mitigation measures are driving disease transmission and finch declines remains uncertain and requires further investigation. However, key precautionary actions for supplementary food providers to mitigate negative effects should be to follow the existing best-practice guidance for disease prevention and control^23,98^, such as that produced by Garden Wildlife Health (available from www.gardenwildlifehealth.org), and to report sightings of ill health in garden birds to the same website so that a veterinary diagnosis can be reached, enabling provision of further targeted guidance and supporting the monitoring and research effort.

## Supplementary Information

Supplementary Information 1.

Supplementary Information 2.

## Data Availability

All data generated or analysed during this study are included in this published article and its Supplementary Information files.

## References

[1] Gregory RD, van Strien A (2010). Wild bird indicators: Using composite population trends of birds as measures of environmental health. Ornithol. Sci..

[2] Cox DTC, Gaston KJ (2016). Urban bird feeding: Connecting people with nature. PLoS ONE.

[3] Anderson RM, May RM (1979). Population biology of infectious diseases: Part I. Nature.

[4] Keesing F (2010). Impacts of biodiversity on the emergence and transmission of infectious diseases. Nature.

[5] Smith KF, Acevedo-Whitehouse K, Pedersen AB (2009). The role of infectious diseases in biological conservation. Anim. Conserv..

[6] Han BA, Kramer AM, Drake JM (2016). Global patterns of zoonotic disease in mammals. Trends Parasitol..

[7] Estrada-Peña A, Ostfeld RS, Peterson AT, Poulin R, de la Fuente J (2014). Effects of environmental change on zoonotic disease risk: An ecological primer. Trends Parasitol..

[8] Daszak P, Cunningham AA, Hyatt AD (2000). Emerging infectious diseases of wildlife–threats to biodiversity and human health. Science.

[9] Pedersen AB, Jones KE, Nunn CL, Altizer S (2007). Infectious diseases and extinction risk in wild mammals. Conserv. Biol..

[10] Atkinson CT, Samuel MD (2010). Avian malaria *Plasmodium relictum* in native Hawaiian forest birds: Epizootiology and demographic impacts on àapapane *Himatione sanguinea*. J. Avian Biol..

[11] George TL (2015). Persistent impacts of West Nile virus on North American bird populations. Proc. Natl. Acad. Sci. USA..

[12] Dhondt AA, Tessaglia DL, Slothower RL (1998). Epidemic mycoplasmal conjunctivitis in house finches from Eastern North America. J. Wildl. Dis..

[13] Monterroso P (2016). Disease-mediated bottom-up regulation: An emergent virus affects a keystone prey, and alters the dynamics of trophic webs. Sci. Rep..

[14] Cheng TL (2021). The scope and severity of white-nose syndrome on hibernating bats in North America. Conserv. Biol..

[15] Rushton SP (2006). Disease threats posed by alien species: The role of a poxvirus in the decline of the native red squirrel in Britain. Epidemiol. Infect..

[16] Scheele BC (2019). Amphibian fungal panzootic causes catastrophic and ongoing loss of biodiversity. Science.

[17] Bradley CA, Altizer S (2007). Urbanization and the ecology of wildlife diseases. Trends Ecol. Evol..

[18] Murray MH (2019). City sicker? A meta-analysis of wildlife health and urbanization. Front. Ecol. Environ..

[19] Giraudeau M, Mousel M, Earl S, McGraw K (2014). Parasites in the city: Degree of urbanization predicts poxvirus and coccidian infections in house finches (*Haemorhous mexicanus*). PLoS ONE.

[20] Shutt JD, Lees AC (2021). Killing with kindness: Does widespread generalised provisioning of wildlife help or hinder biodiversity conservation efforts?. Biol. Conserv..

[21] Van Doren BM (2021). Human activity shapes the wintering ecology of a migratory bird. Glob. Chang. Biol..

[22] Plummer KE, Risely K, Toms MP, Siriwardena GM (2019). The composition of British bird communities is associated with long-term garden bird feeding. Nat. Commun..

[23] Lawson, B. *et al.* Health hazards to wild birds and risk factors associated with anthropogenic food provisioning. *Philos. Trans. R. Soc. B Biol. Sci.* **373**, 20170091 (2018). 10.1098/rstb.2017.0091 PMC5882997 29531146

[24] Galbraith JA, Stanley MC, Jones DN, Beggs JR (2017). Experimental feeding regime influences urban bird disease dynamics. J. Avian Biol..

[25] Siriwardena GM (2007). The effect of supplementary winter seed food on breeding populations of farmland birds: Evidence from two large-scale experiments. J. Appl. Ecol..

[26] Kubasiewicz LM, Bunnefeld N, Tulloch AIT, Quine CP, Park KJ (2016). Diversionary feeding: An effective management strategy for conservation conflict?. Biodivers. Conserv..

[27] Lawson B (2011). A clonal strain of *Trichomonas gallinae* is the aetiologic agent of an emerging avian epidemic disease. Infect. Genet. Evol..

[28] Robinson RA (2010). Emerging infectious disease leads to rapid population declines of common British birds. PLoS ONE.

[29] Forrester, D. J. & Foster, G. W. Trichomonosis. In: *Parasitic Diseases of Wild Birds* 120–153 (Wiley-Blackwell, 2008).

[30] Lawson B (2011). Evidence of spread of the emerging infectious disease, finch trichomonosis, by migrating birds. EcoHealth.

[31] Lawson B (2012). The emergence and spread of finch trichomonosis in the British Isles. Philos. Trans. R. Soc. B Biol. Sci..

[32] Woodward, I. D. *et al. BirdTrends 2020: Trends in numbers, breeding success and survival for UK breeding birds. Research Report 732. BTO, Thetford.* (2020).

[33] Enoksson B (1988). Age- and sex-related differences in dominance and foraging behaviour of nuthatches *Sitta europaea*. Anim. Behav..

[34] Tarvin KA, Woolfenden GE (1997). Patterns of dominance and aggressive behavior in blue jays at a feeder. Condor.

[35] Brittingham MC, Temple SA (1992). Use of winter feeders by black-capped chickadees. Wildl. Soc..

[36] Woodward I (2020). Population estimates of birds in Great Britain and the United Kingdom. Br. Birds.

[37] Musgrove AJ (2013). Population estimates of birds in Great Britain and the United Kingdom. Br. Birds.

[38] Wernham, C. *et al. The Migration Atlas: Movements of the Birds of Britain and Ireland*. (T & AD Poyser, 2002).

[39] Main IG (2000). The partial migration of Fennoscandian Greenfinches *Carduelis chloris*. Ringing Migr..

[40] Lack, P. C. *The Atlas of Wintering Birds in Britain and Ireland*. (T. & A.D. Poyser, 1986).

[41] Robinson, R. A. BirdFacts: profiles of birds occurring in Britain & Ireland. *BTO, Thetford* (2005). Available at: http://www.bto.org/birdfacts. Accessed: 15 May 2022.

[42] Tratalos J (2007). Bird densities are associated with household densities. Glob. Chang. Biol..

[43] Gregory RD (1999). Broad-scale habitat use of sparrows, finches and buntings in Britain. Die Vogelwelt.

[44] Newton, I. *Finches. New Naturalist Series, Volume: 55*. (HarperCollins, 1972).

[45] Robinson RA, Baillie SR, Crick HQP (2007). Weather-dependent survival: Implications of climate change for passerine population processes. Ibis..

[46] Crick HQP (1992). A bird-habitat coding system for use in Britain and Ireland incorporating aspects of land-management and human activity. Bird Study.

[47] Davies ZG (2009). A national scale inventory of resource provision for biodiversity within domestic gardens. Biol. Conserv..

[48] Balmer, D. E. *et al. Bird Atlas 2007–11: The breeding and wintering birds of Britain and Ireland*. (BTO Books, 2013).

[49] Lawson B (2010). Epidemiology of salmonellosis in garden birds in England and Wales, 1993 to 2003. EcoHealth.

[50] Svensson, L. *Identification guide to European passerines, *4th edition. (BTO, 1992).

[51] Jenni, L. & Winkler, R. *Moult and ageing of European passerines*, 2nd edition. (Helm, 2020).

[52] Baillie SR (2001). The contribution of ringing to the conservation and management of bird populations: A review. Ardea.

[53] Kéry M, Schaub M (2012). Bayesian Population Analysis using WinBUGS: A hierarchical perspective.

[54] R Core Team. R: A language and environment for statistical computing. (2020).

[55] Plummer, M. JAGS: A program for analysis of Bayesian graphical models using Gibbs sampling. in *Proceedings of the 3rd International Workshop on Distributed Statistical Computing (DSC 2003)* (eds. Hornik, K., Leisch, F. & Zeileis, A.) (2003).

[56] Su, Y.-S. & Yajima, M. R2jags: Using R to Run ‘JAGS’. R package version 0.6–1. (2020).

[57] Robinson RA, Morrison CA, Baillie SR (2014). Integrating demographic data: Towards a framework for monitoring wildlife populations at large spatial scales. Methods Ecol. Evol..

[58] Newson SE, Evans KL, Noble DG, Greenwood JJD, Gaston KJ (2008). Use of distance sampling to improve estimates of national population sizes for common and widespread breeding birds in the UK. J. Appl. Ecol..

[59] Newson SE, Massimino D, Johnston A, Baillie SR, Pearce-Higgins JW (2013). Should we account for detectability in population trends?. Bird Study.

[60] Crick HQP, Baillie SR, Leech DI (2003). The UK Nest Record Scheme: its value for science and conservation. Bird Study.

[61] Abadi F, Gimenez O, Arlettaz R, Schaub M (2010). An assessment of integrated population models: Bias, accuracy, and violation of the assumption of independence. Ecology.

[62] Plard F, Turek D, Grüebler MU, Schaub M (2019). IPM2: Toward better understanding and forecasting of population dynamics. Ecol. Monogr..

[63] Weegman MD, Arnold TW, Clark RG, Schaub M (2021). Partial and complete dependency among data sets has minimal consequence on estimates from integrated population models. Ecol. Appl..

[64] Koons DN, Iles DT, Schaub M, Caswell H (2016). A life-history perspective on the demographic drivers of structured population dynamics in changing environments. Ecol. Lett..

[65] Koons DN, Arnold TW, Schaub M (2017). Understanding the demographic drivers of realized population growth rates. Ecol Appl..

[66] Caswell, H. *Matrix population models: Construction, analysis and interpretation*. (Sinauer Associates, 2001).

[67] Stubben C, Milligan B (2007). Estimating and analyzing demographic models using the popbio package in R. J. Stat. Softw..

[68] Stanbury A (2021). The status of our bird populations: The fifth Birds of Conservation Concern in the United Kingdom, Channel Islands and Isle of Man and second IUCN Red List assessment of extinction risk for Great Britain. Br. Birds.

[69] Lehikoinen A, Lehikoinen E, Valkama J, Väisänen RA, Isomursu M (2013). Impacts of trichomonosis epidemics on greenfinch *Chloris chloris* and chaffinch *Fringilla coelebs* populations in Finland. Ibis.

[70] PECBMS. EBCC/BirdLife/RSPB/CSO’ Pan-European Common Bird Monitoring Scheme. (2021). Available at: https://pecbms.info/. (Accessed: 14th July 2022)

[71] Keller, V. *et al. European Breeding Bird Atlas 2: Distribution, Abundance and Change*. (European Bird Census Council and Lynx Edicions, 2020).

[72] Rijks JM (2019). Trichomonosis in greenfinches (*Chloris chloris*) in the Netherlands 2009–2017: A concealed threat. Front. Vet. Sci..

[73] Boele, A. *et al. Broedvogels in Nederland in 2020. Sovonrapport 2022/05.* (Sovon Vogelonderzoek Nederland, Nijmegen., 2022).

[74] Jones, D. *The Birds at My Table: Why We Feed Wild Birds and Why It Matters*. (Cornell University Press, 2018).

[75] Pennycott TW (1998). Causes of death of wild birds of the family fringillidae in Britain. Vet. Rec..

[76] Bouwman KM, Hawley DM (2010). Sickness behaviour acting as an evolutionary trap? Male house finches preferentially feed near diseased conspecifics. Biol. Lett..

[77] Lawson B (2011). Acute necrotising pneumonitis associated with *Suttonella ornithocola* infection in tits (Paridae). Vet. J..

[78] Clewley GD, Robinson RA, Clark JA (2018). Estimating mortality rates among passerines caught for ringing with mist nets using data from previously ringed birds. Ecol. Evol..

[79] Francis ML (2018). Effects of supplementary feeding on interspecific dominance hierarchies in garden birds. PLoS ONE.

[80] Wojczulanis-Jakubas K, Kulpińska M, Minias P (2015). Who bullies whom at a garden feeder? Interspecific agonistic interactions of small passerines during a cold winter. J. Ethol..

[81] Cramp, S. *Handbook of the Birds of Europe, the Middle East and North Africa. Volume VIII: Crows to Finches.* (Oxford University Press, 1994).

[82] Brook BW, Bradshaw CJA (2006). Strength of evidence for density dependence in abundance time series of 1198 species. Ecology.

[83] Hochachka WM, Dhondt AA (2000). Density-dependent decline of host abundance resulting from a new infectious disease. Proc. Natl. Acad. Sci. USA..

[84] Hochachka WM, Dobson AP, Hawley DM, Dhondt AA (2021). Host population dynamics in the face of an evolving pathogen. J. Anim. Ecol..

[85] Chi JF (2013). The finch epidemic strain of *Trichomonas gallinae *is predominant in British non-passerines. Parasitology.

[86] Orros ME, Fellowes MDE (2015). Wild bird feeding in an urban area: Intensity, economics and numbers of individuals supported. Acta Ornithol..

[87] Dirren S, Borel S, Wolfrum N, Korner-Nievergelt F (2022). *Trichomonas gallinae* infections in the naïve host *Montifringilla nivalis subsp nivalis*. J. Ornithol..

[88] Tulloch AIT, Possingham HP, Joseph LN, Szabo J, Martin TG (2013). Realising the full potential of citizen science monitoring programs. Biol. Conserv..

[89] Silvertown, J., Buesching, C., Jacobson, S. & Rebelo, T. Citizen science and nature conservation. in *Key Topics in Conservation Biology 2* (eds. Macdonald, D. W. & Willis, K. J.) 127–142 (John Wiley & Sons, 2013).

[90] Dickinson JL, Zuckerberg B, Bonter DN (2010). Citizen science as an ecological research tool: Challenges and benefits. Annu. Rev. Ecol. Evol. Syst..

[91] Baillie SR, Wernham CV, Clark JA (1999). Development of the British and Irish ringing scheme and its role in conservation biology. Ringing Migr..

[92] Greenwood JJD (2007). Citizens, science and bird conservation. J. Ornithol..

[93] Horns JJ, Adler FR, Şekercioğlu ÇH (2018). Using opportunistic citizen science data to estimate avian population trends. Biol. Conserv..

[94] Ryan RL, Kaplan R, Grese RE (2001). Predicting volunteer commitment in environmental stewardship programmes. J. Environ. Plan. Manag..

[95] Maund PR (2020). What motivates the masses: Understanding why people contribute to conservation citizen science projects. Biol. Conserv..

[96] Martin VY, Greig EI (2019). Young adults’ motivations to feed wild birds and influences on their potential participation in citizen science: An exploratory study. Biol. Conserv..

[97] Cox DTC, Gaston KJ (2018). Human–nature interactions and the consequences and drivers of provisioning wildlife philos. Philos. Trans. R. Soc. B Biol. Sci..

[98] Murray MH, Becker DJ, Hall RJ, Hernandez SM (2016). Wildlife health and supplemental feeding: A review and management recommendations. Biol. Conserv..

[99] Rocha G, Quillfeldt P (2015). Effect of supplementary food on age ratios of European turtle doves (*Streptopelia turtur L.*). Anim. Biodivers. Conserv..

