# Supplementary material for: Habitat-use influences severe disease-mediated population declines in two of the most common garden bird species in Great Britain
Source: Sci Rep. 2022 Sep 5;12:15055. doi: 10.1038/s41598-022-18880-8 (PMC9445085; doi:10.1038/s41598-022-18880-8)
Supplement: Supplementary file 2 — Supplementary Information 2. [file 41598_2022_18880_MOESM2_ESM.docx]

**Habitat-use influences severe disease-mediated population declines in two of the most common garden bird species in Great Britain**

**Hugh J. Hanmer*^1^, Andrew A Cunningham^2^, Shinto K. John^2^, Shaheed K. Magregor^2^, Robert A Robinson^1^, Katharina Seilern-Moy^2^, Gavin M. Siriwardena^1^, Becki Lawson*^2^**

^1^ British Trust for Ornithology, The Nunnery, Thetford, Norfolk, IP24 2PU, UK

^2^ Institute of Zoology, Zoological Society of London, Regent’s Park, London, NW1 4RY, UK

* Corresponding authors [hugh.hanmer@bto.org](mailto:hugh.hanmer@bto.org) [becki.lawson@ioz.ac.uk](mailto:becki.lawson@ioz.ac.uk)

**Supplementary Material**

**Appendix 1 Post mortem examination (PME) methods**

To establish cause of death, a systematic examination of internal body systems was conducted, supported by microbiological, parasitological, histological and molecular diagnostic testing as appropriate, based on macroscopic findings, in submitted carcasses of all species^1^. Finch trichomonosis causes pharyngitis/ingluvitis/oesophagitis: lesions vary in appearance from red discolouration (consistent with hyperaemia/congestion), upper gastrointestinal tract (GIT) wall thickening, cream-tan mucosal discolouration (consistent with necrosis) and serosal adhesions (consistent with fibrinous inflammation)^2^. Seed fragments in the oropharynx, facial plumage staining with seed and saliva, and thin to emaciated body condition are frequently observed. A case definition was employed for finch trichomonosis and other causes of death, largely following^2^: cases were assigned as suspected diagnosis on the basis of characteristic upper GIT macroscopic lesions alone (as described above) and confirmed when *Trichomonas gallinae* was detected through positive culture and/or PCR amplification. Salmonellosis is an important differential diagnosis for necrotic upper GIT lesions in wild passerines; however, the appearance of focal to multifocal/confluent lesions with this disease can typically be differentiated from trichomonosis^1^ and microbiological examination was used to exclude this potential aetiology in suspected finch trichomonosis cases where poor state of carcass preservation did not permit *T. gallinae* detection.

**Appendix 2 Example habitat-specific survival model JAGS and associated R code for chaffinch**

#load required package

library(R2jags)

# Specify model in BUGS/JAGS language

sink("chaff_mr_block3_hab_phi.jags")

cat("

model {

###SURVIVAL RATES: from RINGING DATA

#three block periods of interest with annual survival varying randomly around a block mean – pre-decline, early decline and late decline

# peri-domestic (human) site analyses/records indicated by _h

# rural site analyses/records indicated by _r

# adults analyses/records indicated by _ad

# first-winter (juvenile) analyses/records indicated by _jv

# Define priors

#pre-decline period

for(i in 1:6){

logit(phi_ad_h[i]) = mu.ad_h1 + eps_ad.phi_h[i]

logit(phi_jv_h[i]) = mu.jv_h1 + eps_jv.phi_h[i]

}

#early decline period

for(i in 7:13){

logit(phi_ad_h[i]) = mu.ad_h2 + eps_ad.phi_h[i]

logit(phi_jv_h[i]) = mu.jv_h2 + eps_jv.phi_h[i]

}

#late decline period

for(i in 14:19){

logit(phi_ad_h[i]) = mu.ad_h3 + eps_ad.phi_h[i]

logit(phi_jv_h[i]) = mu.jv_h3 + eps_jv.phi_h[i]

}

# Separate final year due to truncated recovery period

for(i in 20){

logit(phi_ad_h[i]) = mu.ad_h4

logit(phi_jv_h[i]) = mu.jv_h4

}

#model annual variation

for(i in 1:(nyears-1)){

eps_ad.phi_h[i] ~ dnorm(0, tau.phi_h)

eps_jv.phi_h[i] ~ dnorm(0, tau.phi_h)

eps_ad.phi_r[i] ~ dnorm(0, tau.phi_r)

eps_jv.phi_r[i] ~ dnorm(0, tau.phi_r)

}

# peri-domestic priors

tau.phi_h <- 1/(sd.phi_h*sd.phi_h)

sd.phi_h ~ dunif(0,1)

# Block mean survival priors on logit scale

mu.jv_h1 ~ dnorm(0, 0.01)

mu.ad_h1 ~ dnorm(0, 0.01)

mu.jv_h2 ~ dnorm(0, 0.01)

mu.ad_h2 ~ dnorm(0, 0.01)

mu.jv_h3 ~ dnorm(0, 0.01)

mu.ad_h3 ~ dnorm(0, 0.01)

mu.jv_h4 ~ dnorm(0, 0.01)

mu.ad_h4 ~ dnorm(0, 0.01)

# Inverse logit transformation to get mean survival for the results

mean_phi_ad_h1 <-ilogit(mu.ad_h1)

mean_phi_jv_h1 <-ilogit(mu.jv_h1)

mean_phi_ad_h2 <-ilogit(mu.ad_h2)

mean_phi_jv_h2 <-ilogit(mu.jv_h2)

mean_phi_ad_h3 <-ilogit(mu.ad_h3)

mean_phi_jv_h3 <-ilogit(mu.jv_h3)

# Calculate changes in survival between periods block periods

dif_phi_jv_h12 <- mean_phi_jv_h2 – mean_phi_jv_h1 #diff 1 to 2 ie pre-decline to early decline

dif_phi_ad_h12 <- mean_phi_ad_h2 – mean_phi_ad_h1

dif_phi_jv_h13 <- mean_phi_jv_h3 – mean_phi_jv_h1 #diff 1 to 3 ie pre-decline to late decline

dif_phi_ad_h13 <- mean_phi_ad_h3 – mean_phi_ad_h1

dif_phi_jv_h23 <- mean_phi_jv_h3 – mean_phi_jv_h2 #diff 2 to 3 ie early-decline to late decline

dif_phi_ad_h23 <- mean_phi_ad_h3 – mean_phi_ad_h2

#rural priors

for(i in 1:6){

logit(phi_ad_r[i]) = mu.ad_r1 + eps_ad.phi_r[i]

logit(phi_jv_r[i]) = mu.jv_r1 + eps_jv.phi_r[i]

}

for(i in 7:13){

logit(phi_ad_r[i]) = mu.ad_r2 + eps_ad.phi_r[i]

logit(phi_jv_r[i]) = mu.jv_r2 + eps_jv.phi_r[i]

}

for(i in 14:19){

logit(phi_ad_r[i]) = mu.ad_r3 + eps_ad.phi_r[i]

logit(phi_jv_r[i]) = mu.jv_r3 + eps_jv.phi_r[i]

}

for(i in 20){

logit(phi_ad_r[i]) = mu.ad_r4

logit(phi_jv_r[i]) = mu.jv_r4

}

tau.phi_r <- 1/(sd.phi_r*sd.phi_r)

sd.phi_r ~ dunif(0,1)

mu.jv_r1 ~ dnorm(0, 0.01)

mu.ad_r1 ~ dnorm(0, 0.01)

mu.jv_r2 ~ dnorm(0, 0.01)

mu.ad_r2 ~ dnorm(0, 0.01)

mu.jv_r3 ~ dnorm(0, 0.01)

mu.ad_r3 ~ dnorm(0, 0.01)

mu.jv_r4 ~ dnorm(0, 0.01)

mu.ad_r4 ~ dnorm(0, 0.01)

mean_phi_ad_r1 <-ilogit(mu.ad_r1)

mean_phi_jv_r1 <-ilogit(mu.jv_r1)

mean_phi_ad_r2 <-ilogit(mu.ad_r2)

mean_phi_jv_r2 <-ilogit(mu.jv_r2)

mean_phi_ad_r3 <-ilogit(mu.ad_r3)

mean_phi_jv_r3 <-ilogit(mu.jv_r3)

dif_phi_jv_r12 <- mean_phi_jv_r2 - mean_phi_jv_r1 #diff 1 to 2

dif_phi_ad_r12 <- mean_phi_ad_r2 - mean_phi_ad_r1

dif_phi_jv_r13 <- mean_phi_jv_r3 - mean_phi_jv_r1 #diff 1 to 3

dif_phi_ad_r13 <- mean_phi_ad_r3 - mean_phi_ad_r1

dif_phi_jv_r23 <- mean_phi_jv_r3 - mean_phi_jv_r2 #diff 2 to 3

dif_phi_ad_r23 <- mean_phi_ad_r3 - mean_phi_ad_r2

# Reported recovery probabilities

#shared sd and tau but separate habitat specific eps for adults and first-winters

for ( i in 1:(nyears)){

logit(p_ad_h[i]) <- p_ad.mean_h + eps_ad_h[i]

logit(p_jv_h[i]) <- p_jv.mean_h + eps_jv_h[i]

eps_ad_h[i] ~ dnorm(0, tau.p_h)

eps_jv_h[i] ~ dnorm(0, tau.p_h)

logit(p_ad_r[i]) <- p_ad.mean_r + eps_ad_r[i]

logit(p_jv_r[i]) <- p_jv.mean_r + eps_jv_r[i]

eps_ad_r[i] ~ dnorm(0, tau.p_r)

eps_jv_r[i] ~ dnorm(0, tau.p_r)

}

#peri-domestic p

p_ad.mean_h ~ dnorm(0, 0.01)

p_jv.mean_h ~ dnorm(0, 0.01)

tau.p_h <- 1/pow(sd.p_h,2) #

sd.p_h ~ dunif(0.001,100)

#rural p

p_ad.mean_r ~ dnorm(0, 0.01)

p_jv.mean_r ~ dnorm(0, 0.01)

tau.p_r <- 1/pow(sd.p_r,2)

sd.p_r ~ dunif(0.001,100)

#Inverse logit transformation to get mean p

pmean_ad_h<- ilogit(p_ad.mean_h)

pmean_jv_h<- ilogit(p_jv.mean_h)

pmean_ad_r<- ilogit(p_ad.mean_r)

pmean_jv_r<- ilogit(p_jv.mean_r)

# Define the likelihood

for ( i in 1:nyears){

#peri-domestic likelihoods

r_ad_h[i,1:(nyears+1)] ~ dmulti(q_ad_h[i,],N_ad_h[i])

r_jv_h[i,1:(nyears+1)] ~ dmulti(q_jv_h[i,],N_jv_h[i])

#rural likelihoods

r_ad_r[i,1:(nyears+1)] ~ dmulti(q_ad_r[i,],N_ad_r[i])

r_jv_r[i,1:(nyears+1)] ~ dmulti(q_jv_r[i,],N_jv_r[i])

}

# Define the cell probabilities of the m-arrays

#Peri-domestic m-arrays

# Define the cell probabilities of the first-winter m-array - code in that from i+2 juv is now an adult

# Main diagonal

for (i in 1:nyears){

q_jv_h[i,i] <- (1-phi_jv_h[i])*p_jv_h[i]

# Further above main diagonal

for (j in (i+2):nyears){

q_jv_h[i,j] <- phi_jv_h[i]*prod(phi_ad_h[(i+1):(j-1)])*(1 -phi_ad_h[j])*p_ad_h[j]

} #j

# Below main diagonal

for (j in 1:(i-1)){

q_jv_h[i,j] <- 0

} #j

} #t

for (i in 1:(nyears-1)){

# One above main diagonal

q_jv_h[i,i+1] <- phi_jv_h[i]*(1-phi_ad_h[i+1])*p_ad_h[i+1]

} #t

# Last column: probability of non-recovery

for (i in 1:nyears){

q_jv_h[i,nyears+1] <- 1-sum(q_jv_h[i,1:nyears])

} #t

# Define the cell probabilities of the adult m-array - adult always the same in standard model

# Main diagonal

for (i in 1:nyears){

q_ad_h[i,i] <- (1-phi_ad_h[i])*p_ad_h[i]

# Above main diagonal

for (j in (i+1):nyears){

q_ad_h[i,j] <- prod(phi_ad_h[i:(j-1)])*(1-phi_ad_h[j])*p_ad_h[j]

} #j

# Below main diagonal

for (j in 1:(i-1)){

q_ad_h[i,j] <- 0

} #j

} #t

# Last column: probability of non-recovery

for (i in 1:nyears){

q_ad_h[i,nyears+1] <- 1-sum(q_ad_h[i,1:nyears])

} #t

# Rural m-arrays

#First-winter m-array cell probabilities

# Main diagonal

for (i in 1:nyears){

q_jv_r[i,i] <- (1-phi_jv_r[i])*p_jv_r[i]

# Further above main diagonal

for (j in (i+2):nyears){

q_jv_r[i,j] <- phi_jv_r[i]*prod(phi_ad_r[(i+1):(j-1)])*(1 -phi_ad_r[j])*p_ad_r[j]

} #j

# Below main diagonal

for (j in 1:(i-1)){

q_jv_r[i,j] <- 0

} #j

} #t

for (i in 1:(nyears-1)){

# One above main diagonal

q_jv_r[i,i+1] <- phi_jv_r[i]*(1-phi_ad_r[i+1])*p_ad_r[i+1]

} #t

# Last column: probability of non-recovery

for (i in 1:nyears){

q_jv_r[i,nyears+1] <- 1-sum(q_jv_r[i,1:nyears])

} #t

# Adult m-array cell probilities

# Main diagonal

for (i in 1:nyears){

q_ad_r[i,i] <- (1-phi_ad_r[i])*p_ad_r[i]

# Above main diagonal

for (j in (i+1):nyears){

q_ad_r[i,j] <- prod(phi_ad_r[i:(j-1)])*(1-phi_ad_r[j])*p_ad_r[j]

} #j

# Below main diagonal

for (j in 1:(i-1)){

q_ad_r[i,j] <- 0

} #j

} #t

# Last column: probability of non-recovery

for (i in 1:nyears){

q_ad_r[i,nyears+1] <- 1-sum(q_ad_r[i,1:nyears])

} #t

}

",fill = TRUE)

sink()

# Rural

# Define the cell probabilities of the juvenile m-array - issue that from i+2 juv is now an adult - needs to be coded in

# Main diagonal

for (i in 1:nyears){

q_jv_r[i,i] <- (1-phi_jv_r[i])*p_jv_r[i]

# Further above main diagonal

for (j in (i+2):nyears){

q_jv_r[i,j] <- phi_jv_r[i]*prod(phi_ad_r[(i+1):(j-1)])*(1-phi_ad_r[j])*p_ad_r[j]

} #j

# Below main diagonal

for (j in 1:(i-1)){

q_jv_r[i,j] <- 0

} #j

} #t

for (i in 1:(nyears-1)){

# One above main diagonal

q_jv_r[i,i+1] <- phi_jv_r[i]*(1-phi_ad_r[i+1])*p_ad_r[i+1]

} #t

# Last column: probability of non-recovery

for (i in 1:nyears){

q_jv_r[i,nyears+1] <- 1-sum(q_jv_r[i,1:nyears])

} #t

# Define the cell probabilities of the adult m-array - adult always the phi_adme in standard model

# Main diagonal

for (i in 1:nyears){

q_ad_r[i,i] <- (1-phi_ad_r[i])*p_ad_r[i]

# Above main diagonal

for (j in (i+1):nyears){

q_ad_r[i,j] <- prod(phi_ad_r[i:(j-1)])*(1-phi_ad_r[j])*p_ad_r[j]

} #j

# Below main diagonal

for (j in 1:(i-1)){

q_ad_r[i,j] <- 0

} #j

} #t

# Last column: probability of non-recovery

for (i in 1:nyears){

q_ad_r[i,nyears+1] <- 1-sum(q_ad_r[i,1:nyears])

} #t

}

",fill = TRUE)

sink()

## Set up model to run

#read in ringing and recovery data

jags.data<-readRDS(file="supplementary_datasets/jags.data_chaff_mr.rds")

# Equivalent greenfinch file is named "jags.data_grefi_mr.rds"

# Set initial values – see JAGS code for explanations

jags.inits<-

function(){list(

### SURVIVAL (phi) AND RECOVERY (p) PROBABILITIES

#peri-domestic

mu.jv_h1=rnorm(1,0, 0.01),

mu.jv_h2=rnorm(1,0, 0.01),

mu.jv_h3=rnorm(1,0, 0.01),

mu.ad_h1=rnorm(1,0, 0.01),

mu.ad_h2=rnorm(1,0, 0.01),

mu.ad_h3=rnorm(1,0, 0.01),

p_ad.mean_h=runif(1, 0, 1),

p_jv.mean_h=runif(1, 0, 1),

mu.jv_r1=rnorm(1,0, 0.01),

mu.jv_r2=rnorm(1,0, 0.01),

mu.jv_r3=rnorm(1,0, 0.01),

mu.ad_r1=rnorm(1,0, 0.01),

mu.ad_r2=rnorm(1,0, 0.01),

mu.ad_r3=rnorm(1,0, 0.01),

p_ad.mean_r=runif(1, 0, 1),

p_jv.mean_r=runif(1, 0, 1)

)}

#parameters to keep for output – see JAGS code for explanations

parameters <- c(

#peri-domestic

'phi_ad_h','phi_jv_h','mean_phi_ad_h1','mean_phi_ad_h2','mean_phi_ad_h3',

'mean_phi_jv_h1','mean_phi_jv_h2','mean_phi_jv_h3',

'dif_phi_jv_h12','dif_phi_ad_h12','dif_phi_jv_h13','dif_phi_ad_h13','dif_phi_jv_h23','dif_phi_ad_h23',

'p_ad_h','p_jv_h','pmean_ad_h','pmean_jv_h',

#rural

'phi_ad_r','phi_jv_r','mean_phi_ad_r1','mean_phi_ad_r2','mean_phi_ad_r3',

'mean_phi_jv_r1','mean_phi_jv_r2','mean_phi_jv_r3',

'dif_phi_jv_r12','dif_phi_ad_r12','dif_phi_jv_r13','dif_phi_ad_r13','dif_phi_jv_r23','dif_phi_ad_r23',

'p_ad_r','p_jv_r','pmean_ad_r','pmean_jv_r')

# Call JAGS from R and run model - run in parallel to reduce model runtime

chaff_mr_block3_hab_phi <- jags.parallel(data=jags.data, inits=jags.inits, parameters.to.save=parameters, model.file="chaff_mr_block3_hab_phi.jags", n.chains = 3, n.thin = 10, n.iter = 100000, n.burnin = 50000,n.cluster = 3, working.directory = getwd())

#save image

save.image("output/chaff_mr_block3_hab_phi.RData")

**Appendix 3 Integrated Population Model (IPM) structure and fitting**

**Integrated Population Model (IPM) structure and data sources**

We combined data from three, national, long-term citizen science monitoring programmes operated by the British Trust for Ornithology (BTO) between 2000 and 2019 into an integrated population model, largely following Robinson *et al.*^3^: 1) population trend data from the BTO/RSPB/JNCC Breeding Bird Survey (BBS)^4,5^; 2) mark-recovery survival data from the BTO bird ringing scheme for adult and first winter birds across all habitats^3,6^; and 3) nest productivity and survival data from the BTO Nest Record Scheme (NRS; Crick *et al.*^7^).

**Population model**

Birds have a multi-year life cycle, where the breeding population $(N)$ in any given year $(t)$ comprises adult individuals that have survived from the previous year to breed again $(N_{t})$ and young birds hatched the previous year that have survived to be recruited into the following year’s breeding population $(Nr)$:

1. $N_{t}= N_{r,t-1}+N_{a,t}$

These processes are generally considered to be stochastic and can be represented by Poisson and binomial processes respectively:

1. $N_{r,t}\sim Po(N_{t}*v_{t})$
2. $N_{a,t}\sim Bin(\varphi_{ad,t},N_{t-1})$

Where $v_{t}$ is the mean number of young birds per adult individual recruiting into the following years adult population and $\varphi_{ad,t}$ is the probability of existing adult individuals surviving from the previous year to the next. Both chaffinch and greenfinch typically breed in their first year, so were assumed to do so (Cramp^8^; although see later in relation to ρ). For simplicity, only the female half of the population was modelled on the assumption of an even sex-ratio in the whole population. As the drivers of population change were of primary interest, the finite rate of population change $(\lambda)$ in year $t$was then derived as the ratio of total population sizes in consecutive years $t$ and $t-1$:

1. $\lambda_{t}=\frac{N_{t}}{N_{t-1}}$

As the true annual national population sizes for most wild animals including our study species were unknown $(N_{t})$, we instead used annual population size indices derived from the line transect based BBS survey^4^. We assumed that the underlying population size $(N_{t})$ under a steady state-space model was log-normally related to the observed population indices in a given year $(y_{t})$. These indices were estimated using generalized linear models (GLM) where the number of birds recorded at each site was assumed to be Poisson-distributed and a function of two fixed factors: site and year, with the estimated year terms taken as an index of population size $(y_{t})$. Uncertainty was represented by the associated standard errors for these count estimates $(\sigma_{t})$:

1. $ln(y_{t})\sim N(\ln\left( N_{t} \right),\sigma_{t}^{2})$

As no abundance data are available for 2001 due to access restrictions following a widespread outbreak of foot-and-mouth disease^9^, population change between 2000-2001 and 2001-2002 could not be directly calculated and so was treated as a missing value with the standard error term for the 2001 index value taken to be the mean value for the whole study period.

The recruitment parameter $\nu$ was split into productivity (averaged over all individuals) and survival from independence to the start of the breeding season. Both of these factors are expected to be influenced by various, potentially different, ecological factors.

Productivity is a function of fecundity (number of eggs laid), the number of those that survive to hatch (hatching success) and the number of resulting chicks that subsequently fledge (fledging success). Brood size $(B_{t})$ was taken to be the maximum recorded brood size recorded by the BTO Nest Record Scheme (NRS) and partial clutch and brood losses were not captured directly. Brood size $(B_{t})$ was modelled using an exponentially weighted Poisson distribution (EWP), which provides an empirical fit to under-dispersed discrete data^10^. To quantify nest survival, we used data from nests visited multiple times by participants in the BTO NRS, who record the date and nest contents on each visit made, and from which the daily probability of failure can be estimated^7^. As the date on which the first egg is laid is not accurately known for most nests, we modelled average daily nest survival rates within each of the egg incubation and chick-rearing stages. Overall egg $(\varphi_{egg,t})$ and young stage $(\varphi_{yng,t})$survival probabilities were calculated based on average incubation and fledging periods for the two species (data taken from Robinson^11^).

Individual age-specific survival probability across all habitats was estimated using standard multinomial models of mark-recovery data^12^. Within the same model, annual survival (*φ_t_*) was estimated, for first-winter birds, from fledging to 30^th^ June the following calendar year ($\varphi_{fw,t}$) and, for adults, from 1^st^ July to the following 30^th^ June ${(\varphi}_{ad,t})$. Birds of uncertain age at the time of ringing were excluded. Age-specific survival was modelled using period-specific means$\mu_{h,p}$ with annual residuals $\varepsilon\sim N(0, {\sigma^{2}}_{p})$. Furthermore, to assess whether survival in either age class had declined around the identified population change points (the outbreak of trichomonosis in Britain in 2005/06 and at the subsequent chaffinch population decline around 2013/14), the mean static survival probability with associated annual residuals ε[t] was modelled separately for each of the three identified periods (2000-2005, pre-decline; 2006-2012, early decline; 2013-2019, late decline). The difference between the mean survival for these three periods was then calculated. Note that, as survival for the final year of data (birds ringed in 2019) cannot be fully assessed due to the truncated potential reporting period, this was not included in the mean periods.

The reported number of individuals ringed in a given year’s cohort that are recovered in year $t$ is a product of the age-specific annual survival probabilities $(\varphi)$ from the period of ringing up to $t-1$, the probability of mortality in year $t (1-\varphi_{t})$, and the probability that, having died, a ringed individual is found and reported ($p_{t}$). This reporting probability was also modelled as an age-specific mean $\mu_{a}$ over the entire study period, with annual residuals $\varepsilon_{t}\sim N(0, {\sigma^{2}}_{a})$.

There are three further demographic aspects that contribute to recruitment $\upsilon$ that cannot be easily measured for these study species due to lack of data, namely the number of broods that pairs attempt to raise, post-fledging survival (between fledging and independence: Robinson *et al.*^3^) and breeding propensity. Chaffinches are typically single-brooded, although can occasionally make a second attempts in addition to replacement clutches following early failure, while greenfinches tend to be double brooded^8,11^. Exact breeding propensity, although presumed to be near 100%, is unknown for both of these species, with all individuals expected to start attempting to breed from their second year of life^8^. In addition, few of either species are routinely ringed as nestlings with even fewer subsequently recovered and reported making it difficult to assess survival in the immediate post-fledging period prior to the majority of juvenile birds being ringed^13,14^. All three variables may vary from year to year, so to incorporate uncertainty caused by these poorly measured variables, a scaling parameter ρ was added into the recruitment term, following Robinson *et al.*^3^. As this parameter was not informed directly by data it largely reflected the difference between the observed population size and the estimates from the measured demographic data.

For more details on the population model and associated datasets used in this study see Robinson et al.^3^.

**Fitting the IPM**

Following Robinson et al. ^3^, a state–space model combining an underlying system process describing annual population change (eqn 1) with an observation process (eqn 4) that relates the true population size $(N_{t})$ to the observed counts $(y_{t})$ was used. Recruitment $(v_{t})$ was represented as the product of the brood/clutch size $(B_{t})$, nest survival at the egg ${(\varphi}_{egg,t}^{ep})$ and young stage ${(\varphi}_{yng,t}^{yp})$ and first winter survival $\varphi_{fw}$:

1. $N_{t+1}=0.5N_{t}\rho_{t}\left( B_{t}\varphi_{yng,t}^{ep}\varphi_{yng,t}^{yp} \right)+N_{t}\varphi_{ad,t}$

Where $ep$ and $yp$ are the (fixed) numbers of days that nests contain eggs and nestlings, respectively (using mean periods from BTO NRS data; Robinson^11^), and $\rho$ combines all unmeasured demographic variables. Recruitment is multiplied by 0.5 to account for only considering the female half of the population. Since the true population was unknown in year one and our observed population trajectory $(y)$ is based on an index, $N_{1}$was arbitrarily set to 1000.

The IPM was fitted in a Bayesian paradigm, again following Robinson et al.^3^, combining (generally) uninformative priors on the parameters, with their joint probability density function being calculated by multiplying the likelihoods together. This assumes that the data sources are independent, which is unlikely to be strictly true; previous studies, however, have suggested this is unlikely to bias the results substantially^15^. For mean period survival $(\varphi)$ and overall mean reporting $(p)$ probabilities, we specified dnorm (1, 0.01) priors. As the range of plausible values for mean annual fecundity was limited, a normal prior was specified, with a mean close to the expected mean brood size and unit variance. For the two weighting parameters of the EWP for brood size, we used uniform (0, 5) priors. For other priors see example JAGS code below.

The IPM was fitted in JAGS with a sampling process of 500,000 iterations with three independent chains, a burn-in period of 400,000 iterations and a thinning value of 20 (i.e. 15,000 iterations were sampled). Model convergence across the chains was assessed using the Brooks-Gelman–Rubin statistic $\hat{R}$, with all $\hat{R}$ < 1.05. We also inspected output trace plots to confirm effective burn-in and chain mixing across the full parameter space.

**Example IPM JAGS and associated R code for chaffinch with notes for greenfinch where the models differ**

library(rjags)

sink("chaff_ipm_block3.jags")

cat("

model{

####POPULATION MODEL: SYSTEM PROCESS

# Define the prior for 'number of broods' parameter

nb_mean ~ dunif(0,2) # (0,2) in chaffinch, (0,3) in greenfinch

nb_tau ~ dgamma(0.01,0.01)

# because recursive need to set population in first year

N1[1] <- 0

Na1 ~ dpois(1000)

Na[1] <- round(Na1) # because counts are on log-scale

# The system process: a simple annual stochastic demographic model

for ( i in 1:(nyears-1)){

# rho has to be positive

rho[i] ~ dnorm(nb_mean,nb_tau)

# Number of birds fledged per attempt

Nfledge[i] <- bs_mean[i] * pow((1-dfail_e[i]),12.28 ) * pow((1-dfail_y[i]), 14.12)

# Nfledge[i] <- bs_mean[i] * pow((1-dfail_e[i]),13.70 ) * pow((1-dfail_y[i]), 14.92) for greenfinch

# Number of recruits into breeding population the following year

Nr[i] <- Na[i] * rho[i] * 0.5*Nfledge[i] * phi_jv[i]

# Number of adults surviving

Ns[i] ~ dbin(phi_ad[i],Na[i])

}

# for remaining years add recruits and surviving adults

for ( i in 2:nyears){

N1[i] ~ dpois(Nr[i-1])

Na[i] <- N1[i] + Ns[i-1]

}

# Calculate Population Growth Rate

for ( i in 1:(nyears-1)){

R[i] <- Na[i+1] / Na[i]

}

#### POPULATION MODEL: OBSERVATION PROCESS

# Observation process: adults are a normal sample of those existing

# with sigma based on MLE parameters corrected according to King et al (2008) Bayesian Analysis in Population Ecology.

# y[i] and yse[i] are on log scale so need to transform Na

#Due to foot and month affecting BBS coverage in 2001 (year 2) set prior for that year

yse[2]~dunif(0,50)

for ( i in 1:nyears ) {

obs_tau[i] <- 1 / pow(yse[i],2)

lnNa[i] <- log(Na[i])

y[i] ~ dnorm(lnNa[i], obs_tau[i])

}

###SURVIVAL RATES: from RINGING DATA

#three block periods of interest with annual survival varying randomly around a block mean – pre-decline, early decline and late decline

# adults analyses/records indicated by _ad

# first-winter (juvenile) analyses/records indicated by _jv

# Define priors

#pre-decline period

for(i in 1:6){

logit(phi_jv[i]) = mu.jv1 + eps_jv.phi[i]

logit(phi_ad[i]) = mu.ad1 + eps_ad.phi[i]

}

#early decline period

for(i in 7:13){

logit(phi_jv[i]) = mu.jv2 + eps_jv.phi[i]

logit(phi_ad[i]) = mu.ad2 + eps_ad.phi[i]

}

late decline period

for(i in 14:19){

logit(phi_jv[i]) = mu.jv3 + eps_jv.phi[i]

logit(phi_ad[i]) = mu.ad3 + eps_ad.phi[i]

}

# Separate final year due to truncated recovery period

for(i in 20){

logit(phi_jv[i]) = mu.jv4

logit(phi_ad[i]) = mu.ad4

}

#model annual variation

for ( i in 1:(nyears-1)){

eps_jv.phi[i] ~ dnorm(0, tau.phi)

eps_ad.phi[i] ~ dnorm(0, tau.phi)

}

#block mean survival priors on logit scale

mu.jv1 ~ dnorm(0, 0.01)

mu.ad1 ~ dnorm(0, 0.01)

mu.jv2 ~ dnorm(0, 0.01)

mu.ad2 ~ dnorm(0, 0.01)

mu.jv3 ~ dnorm(0, 0.01)

mu.ad3 ~ dnorm(0, 0.01)

mu.jv4 ~ dnorm(0, 0.01)

mu.ad4 ~ dnorm(0, 0.01)

tau.phi <- 1/(sd.phi*sd.phi)

sd.phi ~ dunif(0,10)

# reported recovery probabilities

#shared sd and tau but seperate eps for ads and juvs

for ( i in 1:(nyears)){

logit(p_jv[i]) <- p_jv.mean + eps_jv[i]

logit(p_ad[i]) <- p_ad.mean + eps_ad[i]

eps_jv[i] ~ dnorm(0, tau.p)

eps_ad[i] ~ dnorm(0, tau.p)

}

p_jv.mean ~ dnorm(0, 0.01)

p_ad.mean ~ dnorm(0, 0.01)

tau.p <- 1/pow(sd.p,2)

sd.p ~ dunif(0.001,100)

# Define the likelihood

for ( i in 1:nyears){

r_ad[i,1:(nyears+1)] ~ dmulti(q_ad[i,],N_ad[i])

r_jv[i,1:(nyears+1)] ~ dmulti(q_jv[i,],N_jv[i])

}

# Define the cell probabilities of the m-arrays

# Define the cell probabilities of the first-winter m-array - code in that from i+2 juv is now an adult

# Main diagonal

for (i in 1:nyears){

q_jv[i,i] <- (1-phi_jv[i])*p_jv[i]

# Further above main diagonal

for (j in (i+2):nyears){

q_jv[i,j] <- phi_jv[i]*prod(phi_ad[(i+1):(j-1)])*(1-phi_ad[j])*p_ad[j]

} #j

# Below main diagonal

for (j in 1:(i-1)){

q_jv[i,j] <- 0

} #j

} #t

for (i in 1:(nyears-1)){

# One above main diagonal

q_jv[i,i+1] <- phi_jv[i]*(1-phi_ad[i+1])*p_ad[i+1]

} #t

# Last column: probability of non-recovery

for (i in 1:nyears){

q_jv[i,nyears+1] <- 1-sum(q_jv[i,1:nyears])

} #t

# Define the cell probabilities of the adult m-array

# Main diagonal

for (i in 1:nyears){

q_ad[i,i] <- (1-phi_ad[i])*p_ad[i]

# Above main diagonal

for (j in (i+1):nyears){

q_ad[i,j] <- prod(phi_ad[i:(j-1)])*(1-phi_ad[j])*p_ad[j]

} #j

# Below main diagonal

for (j in 1:(i-1)){

q_ad[i,j] <- 0

} #j

} #t

# Last column: probability of non-recovery

for (i in 1:nyears){

q_ad[i,nyears+1] <- 1-sum(q_ad[i,1:nyears])

} #t

#### PRODUCTIVITY: BROOD SIZES from RINGING DATA

# Define the priors

for ( i in 1:nyears){

# Use an informative prior for brood size since we have a good idea of what they might be

bs_mean[i] ~ dunif(1 , 6)

}

# two weighting parameters for exponential Poisson

Beta1 ~ dunif(0,5)

Beta2 ~ dunif(0,5)

for ( i in 1:nyears){

for ( k in min_brood:max_brood ){

# use step() to identify whether greater or less than mean brood size

ind[i,k] <- step(k-bs_mean[k])

w_k[i,k] <- ind[i,k] * exp(-1*Beta2*(k-bs_mean[i])) + (1-ind[i,k]) * exp(-1*Beta1*(bs_mean[i]-k))

w[i,k] <- (exp(-1*bs_mean[i])*pow(bs_mean[i],k)*w_k[i,k])/exp(logfact(k))

}

wcap[i]<-sum(w[i,])

}

for ( i in 1:nyears ){

for ( k in min_brood:max_brood ){

f[i,k] <- w[i,k] / wcap[i]

bsize[i,k] ~ dbin(f[i,k],ncases[i])

}

}

#### PRODUCTIVITY: NEST FAILURE RATES from NEST RECORD DATA

# Define the priors

for ( i in 1:nyears ) {

dfail_e[i] ~ dbeta(1,1)

dfail_y[i] ~ dbeta(1,1)

}

# Calculate the likelihoods: note these are daily failure rates

for ( i in 1:erec ){

edays[i] <- egg_ed[i] * etrial[i]

efail[i] ~ dbin(dfail_e[eggyear[i]], edays[i])

}

for ( i in 1:yrec ){

ydays[i] <- yng_ed[i] * ytrial[i]

yfail[i] ~ dbin(dfail_y[yngyear[i]], ydays[i])

}

## Get mean values for demographic variables for the results

# Inverse logit transformations to get mean survival and reported recovery probability

mean_phi_jv1 <- ilogit(mu.jv1)

mean_phi_ad1 <- ilogit(mu.ad1)

mean_phi_jv2 <- ilogit(mu.jv2)

mean_phi_ad2 <- ilogit(mu.ad2)

mean_phi_jv3 <- ilogit(mu.jv3)

mean_phi_ad3 <- ilogit(mu.ad3)

pmean_jv<-ilogit(p_jv.mean)

pmean_ad<-ilogit(p_ad.mean)

# Calculate changes in survival between periods block periods

dif_phi_jv12 <- mean_phi_jv2 - mean_phi_jv1 #diff 1 to 2

dif_phi_ad12 <- mean_phi_ad2 - mean_phi_ad1

dif_phi_jv13 <- mean_phi_jv3 - mean_phi_jv1 #diff 1 to 3

dif_phi_ad13 <- mean_phi_ad3 - mean_phi_ad1

dif_phi_jv23 <- mean_phi_jv3 - mean_phi_jv2 #diff 2 to 3

dif_phi_ad23 <- mean_phi_ad3 - mean_phi_ad2

#overall nest survival rates for plotting

#calulation probability of daily nest survival (1-daily failure)^days exposure i.e. mean number of egg and nestling days in the nest

for ( i in 1:(nyears-1)){

#egg stage nest survial

phi_e[i]<-pow((1-dfail_e[i]),12.28) #13.70 in greenfinch

nestling (young) stage nest survival

phi_y[i]<-pow((1-dfail_y[i]),14.12) #14.92 in greenfinch

#overall nest surivial - may be useful in some analyses

phi_n[i]<-pow((1-dfail_e[i]),12.28 ) * pow((1-dfail_y[i]), 14.12)

}

#get overall mean daily failure egg and young survival

mean_de <- mean(dfail_e)

mean_dy <- mean(dfail_y)

#overall nest stage survival

mean_phi_e <- mean(phi_e)

mean_phi_y <- mean(phi_y)

mean_phi_n <- mean(phi_n) #overall nest survival

#mean brood size

mean_bs<- mean(bs_mean)

#rho

mean_rho<-mean(rho)

}

",fill = TRUE)

sink()

## Set up model to run

# Read in all data - see

jags.data<-readRDS(file="supplementary_datasets/jags.data_chaff_ipm.rds")

# Equivalent greenfinch file is named "jags.data_grefi_ipm.rds"

# Set initial values – see JAGS code for explanations

jags.inits<-

function(){list(

### SURVIVAL (phi) AND RECOVERY (p) PROBABILITIES

mu.jv1=rnorm(1,0, 0.01),

mu.jv2=rnorm(1,0, 0.01),

mu.jv3=rnorm(1,0, 0.01),

mu.jv4=rnorm(1,0, 0.01),

mu.ad1=rnorm(1,0, 0.01),

mu.ad2=rnorm(1,0, 0.01),

mu.ad3=rnorm(1,0, 0.01),

mu.ad4=rnorm(1,0, 0.01),

p_ad.mean=runif(1, 0, 1),

p_jv.mean=runif(1, 0, 1),

### PRODUCTIVITY: BROOD SIZES

bs_mean=rep(3.0,nyears),

Beta1=0.5,

Beta2=1.3,

### PRODUCTIVITY: NEST FAILURE RATES

dfail_e=rep(0.03,nyears),

dfail_y=rep(0.03,nyears),

### POPULATION: SYSTEM PROCESS

nb_mean=1.0,

rho=rep(1.0,nyears-1)

)}

#parameters to keep for output – see JAGS code for explanations

parameters <- c(

#population parameters

'Na','N1','Ns','Nr','y','R',

#annual survival parameters

'phi_ad','phi_jv',

#annual reporting p parameters

'p_ad','p_jv',

#reproductive parameters

'bs_mean','Beta1', 'Beta2','dfail_e','dfail_y',

#rho for everything else

'rho',

#mean values for results

'mean_phi_ad1','mean_phi_ad2','mean_phi_ad3',

'mean_phi_jv1','mean_phi_jv2','mean_phi_jv3',

'pmean_ad','pmean_jv',

'phi_e','phi_y','phi_n',

'mean_bs','mean_de','mean_dy',

'mean_phi_e','mean_phi_y','mean_phi_n',

'mean_rho',

#survival difference between periods parameters

'dif_phi_jv12','dif_phi_ad12','dif_phi_jv13',

'dif_phi_ad13','dif_phi_jv23','dif_phi_ad23')

# Call JAGS from R and run model - run in parallel to reduce model runtime

chaff_ipm_block3 <- jags.parallel(jags.data, jags.inits, parameters, "chaff_ipm_block3.jags",

n.chains = 3, n.thin = 20, n.iter = 500000, n.burnin = 400000,n.cluster= 3,

working.directory = getwd())

#save output

save.image("output/chaff_ipm_block3.RData")


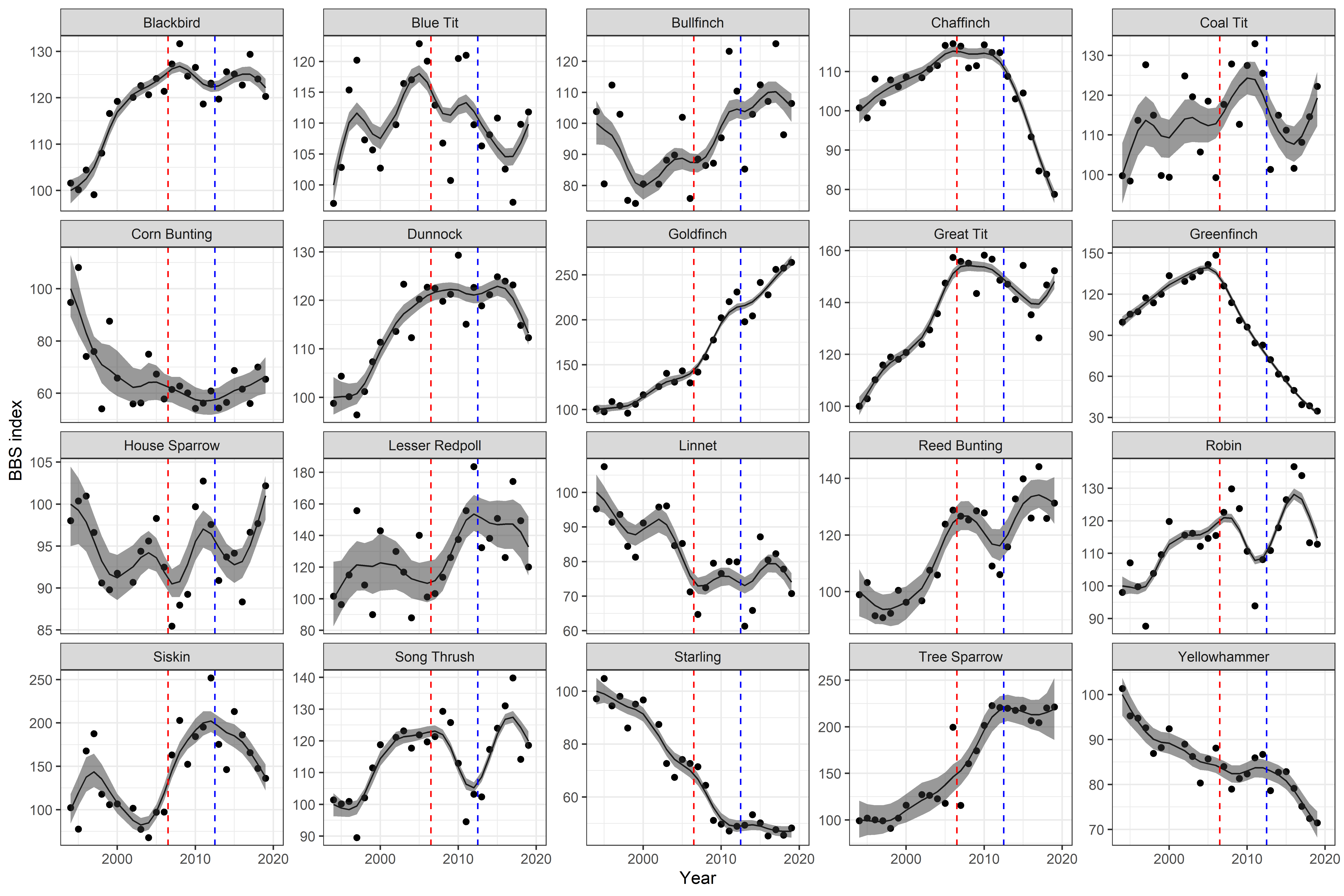
**Appendix 4 Supplementary figures and tables**

*Supplementary Figure 1. Breeding Bird Survey (BBS) trends reproduced from Woodward et al.*^16^ *for a range of garden and farmland using passerines. The vertical red dashed line indicates the start of the Greenfinch decline and Chaffinch plateau period, while the vertical blue dashed line indicates the start of the Chaffinch decline period. Plotted with 85% confidence intervals and indexed from 1994 (the first year of the BBS). Note differing y axes as the indexes are species specific.*

*
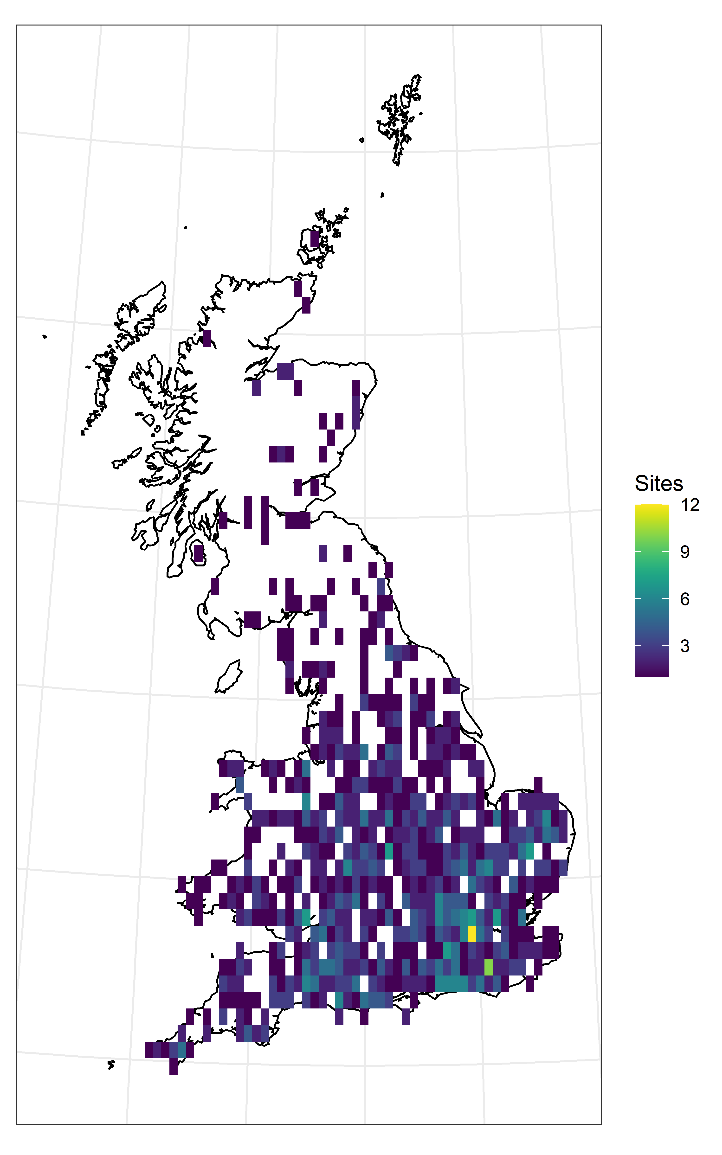
Supplementary Figure 2. Spatial distribution of sites from which carcasses were obtained for post-mortem examination (PME) in this study. Where each site was represented as a 1 km square Ordnance Survey grid reference.*


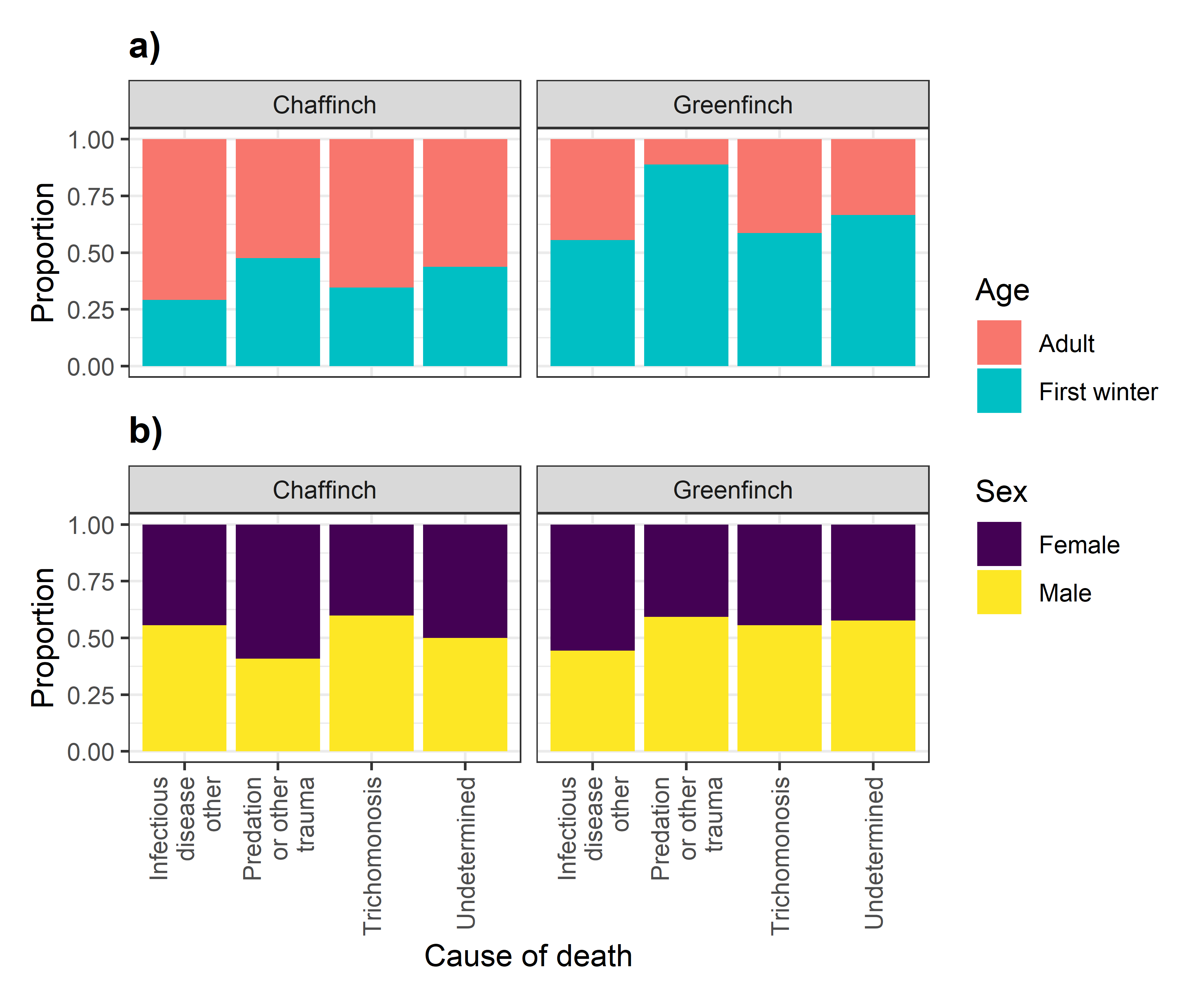


*Supplementary Figure 3. Composition of Chaffinch and Greenfinch examined post-mortem by a) age and b) sex across cause of death categories.*

*
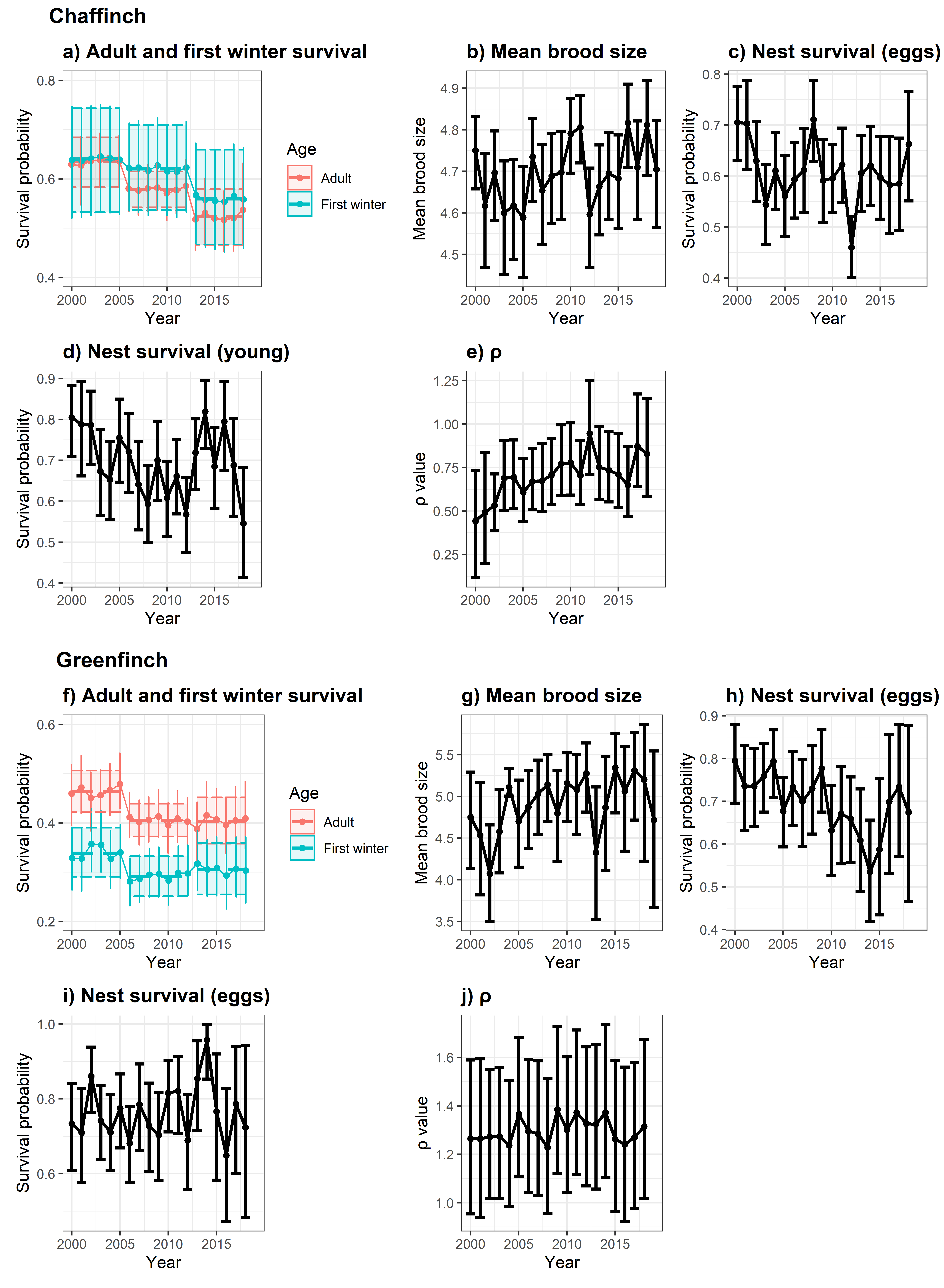
*

*Supplementary Figure 4. Annual demographic rate estimates for a)-e) Chaffinch and f)-j) Greenfinch from species-specific integrated population models. All parameters are per capita rates except for mean brood size, which is a count. Vertical bars show 95% Bayesian confidence intervals (BCI). Horizontal coloured horizontal blocks in a) and f) indicate mean age specific survival for the pre-decline, early decline and late decline periods.*

*Supplementary Table 1. Total passerine species examined post-mortem and diagnosed with trichomonosis from across Great Britain, July 2005-June 2019. Listed in taxonomic order*^17^*.*

| English name | Scientific name | Total examined | Total identified as trichomonosis (suspected and confirmed combined)  **(% overall passerine trichomonosis cases)** |
| --- | --- | --- | --- |
| Jay | *Garrulus glandarius* | 2 | 0 |
| Magpie | *Pica pica* | 8 | 0 |
| Jackdaw | *Coloeus monedula* | 9 | 0 |
| Rook | *Corvus frugilegus* | 5 | 0 |
| Carrion Crow | *Corvus corone* | 12 | 0 |
| Raven | *Corvus corax* | 2 | 0 |
| Waxwing | *Bombycilla garrulus* | 4 | 0 |
| Coal Tit | *Periparus ater* | 14 | 0 |
| Marsh Tit | *Poecile palustris* | 1 | 0 |
| Blue Tit | *Cyanistes caeruleus* | 75 | 0 |
| Great Tit | *Parus major* | 116 | **7 (0.7%)** |
| Sand Martin | *Riparia riparia* | 1 | 0 |
| Barn Swallow | *Hirundo rustica* | 10 | 0 |
| House Martin | *Delichon urbicum* | 9 | 0 |
| Long-tailed Tit | *Aegithalos caudatus* | 10 | 0 |
| Willow Warbler | *Phylloscopus trochilus* | 1 | 0 |
| Chiffchaff | *Phylloscopus collybita* | 3 | 0 |
| Reed Warbler | *Acrocephalus scirpaceus* | 1 | 0 |
| Blackcap | *Sylvia atricapilla* | 10 | 0 |
| Goldcrest | *Regulus regulus* | 11 | 0 |
| Wren | *Troglodytes troglodytes* | 9 | 0 |
| Nuthatch | *Sitta europaea* | 6 | 0 |
| Treecreeper | *Certhia familiaris* | 2 | 0 |
| Starling | *Sturnus vulgaris* | 57 | 0 |
| Song Thrush | *Turdus philomelos* | 30 | 0 |
| Mistle Thrush | *Turdus viscivorus* | 3 | 0 |
| Redwing | *Turdus iliacus* | 12 | 0 |
| Blackbird | *Turdus merula* | 139 | **4 (0.4%)** |
| Fieldfare | *Turdus pilaris* | 3 | 0 |
| Spotted Flycatcher | *Muscicapa striata* | 5 | 0 |
| Robin | *Erithacus rubecula* | 39 | **1 (0.1%)** |
| Redstart | *Phoenicurus phoenicurus* | 1 | 0 |
| Sparrow sp. | *Passer spp.* | 1 | 0 |
| Tree Sparrow | *Passer montanus* | 13 | 0 |
| House Sparrow | *Passer domesticus* | 142 | **21 (2.2%)** |
| Pied Wagtail | *Motacilla alba yarrellii* | 2 | 0 |
| Dunnock | *Prunella modularis* | 75 | **9 (0.9%)** |
| Chaffinch | *Fringilla coelebs* | 385 | **276 (28.7%)** |
| Brambling | *Fringilla montifringilla* | 17 | **13 (1.4%)** |
| Hawfinch | *Coccothraustes coccothraustes* | 4 | **2 (0.2%)** |
| Bullfinch | *Pyrrhula pyrrhula* | 67 | **41 (4.3%)** |
| Greenfinch | *Chloris chloris* | 664 | **500 (52%)** |
| Crossbill | *Loxia spp.* | 1 | 0 |
| Linnet | *Linaria cannabina* | 2 | 0 |
| Lesser Redpoll | *Acanthis cabaret* | 5 | **1 (0.1%)** |
| Goldfinch | *Carduelis carduelis* | 110 | **66 (6.9%)** |
| Siskin | *Spinus spinus* | 67 | **15 (1.6%)** |
| Yellowhammer | *Emberiza citronella* | 16 | **5 (0.5%)** |
| Reed Bunting | *Emberiza schoeniclus* | 3 | 0 |

*Supplementary Table 2. Mean demographic parameter estimates for habitat-specific mark-recapture models and overall Integrated Population Models (IPMs). With Bayesian 95% confidence intervals (BCI).*

| *Species* | *Model* | *Site habitat* | *Adult survival (*$\varphi_{ad}$*)* | | | *First winter survival (*$\varphi_{fw}$*)* | | | *Productivity* | | | $\rho$ |
| --- | --- | --- | --- | --- | --- | --- | --- | --- | --- | --- | --- | --- |
|  |  |  | *Pre-decline*  *period* | *Early decline period* | *Late decline period* | *Pre-decline*  *period* | *Early decline*  *period* | *Late decline*  *period* | *Mean brood size (*$b$*)* | *Mean nest survival (eggs) (*$\varphi_{egg}$*)* | *Mean nest survival (young) (*$\varphi_{egg}$*)* |  |
| *Chaffinch* | *Habitat* | *Peri-domestic* | *0.69 (0.63 - 0.76)* | *0.56 (0.51 - 0.62)* | *0.48 (0.40 - 0.56)* | *0.63 (0.51 - 0.75)* | *0.60 (0.48 - 0.72)* | *0.52 (0.39 - 0.67)* |  |  |  |  |
|  |  | *Rural* | *0.62 (0.54 - 0.70)* | *0.57 (0.51 - 0.63)* | *0.50 (0.42 - 0.59)* | *0.72 (0.58 - 0.84)* | *0.60 (0.48 - 0.74)* | *0.53 (0.38 - 0.70)* |  |  |  |  |
|  | *IPM* | *Overall* | *0.64 (0.59 - 0.69)* | *0.58 (0.54 - 0.62)* | *0.52 (0.46 - 0.58)* | *0.64 (0.53 - 0.74)* | *0.62 (0.54 - 0.71)* | *0.56 (0.47 - 0.66)* | *4.7 (4.6 - 4.7)* | *0.61 (0.59 - 0.63)* | *0.70 (0.67 - 0.72)* | *0.7 (0.59 - 0.82)* |
| *Greenfinch* | *Habitat* | *Peri-domestic* | *0.47 (0.41 - 0.53)* | *0.40 (0.36 - 0.45)* | *0.38 (0.31 - 0.44)* | *0.35 (0.27 - 0.43)* | *0.25 (0.20 - 0.30)* | *0.30 (0.22 - 0.39)* |  |  |  |  |
|  |  | *Rural* | *0.47 (0.39 - 0.54)* | *0.40 (0.34 - 0.46)* | *0.42 (0.34 - 0.51)* | *0.39 (0.29 - 0.49)* | *0.36 (0.28 - 0.44)* | *0.32 (0.23 - 0.42)* |  |  |  |  |
|  | *IPM* | *Overall* | *0.46 (0.42 - 0.51)* | *0.41 (0.37 - 0.44)* | *0.40 (0.36 - 0.45)* | *0.34 (0.29 - 0.39)* | *0.29 (0.25 - 0.33)* | *0.31 (0.26 - 0.36)* | *4.9 (4.6 - 5.2)* | *0.68 (0.64 - 0.71)* | *0.75 (0.71 - 0.78)* | *1.29 (1.12 - 1.48)* |

*Supplementary Table 3. Recovery reporting probability estimates (p) for habitat-specific mark-recapture models and overall IPMs. With Bayesian 95% confidence intervals (BCI).*

| *Species* | *Model* | *Site habitat* | *Reporting probability (*$p$*)* | |
| --- | --- | --- | --- | --- |
|  |  |  | *Adult (*$p_{ad}$*)* | *First winter (*$p_{fw}$*)* |
| *Chaffinch* | *Habitat* | *Peri-domestic* | *0.0062 (0.0053 - 0.0071)* | *0.0102 (0.0077 - 0.0141)* |
|  |  | *Rural* | *0.0042 (0.0037 - 0.0048)* | *0.0082 (0.0059 - 0.0121)* |
|  | *IPM* | *Overall* | *0.0051 (0.0046 - 0.0057)* | *0.0093 (0.0075 - 0.012)* |
| *Greenfinch* | *Habitat* | *Peri-domestic* | *0.0103 (0.0089 - 0.0118)* | *0.0085 (0.0073 - 0.0099)* |
|  |  | *Rural* | *0.0083 (0.0072 - 0.0095)* | *0.0077 (0.0066 - 0.009)* |
|  | *IPM* | *Overall* | *0.0097 (0.0086 - 0.0108)* | *0.0080 (0.0071 - 0.0091)* |

**Supplementary material references**

1. Lawson, B. *et al.* Epidemiology of salmonellosis in garden birds in England and Wales, 1993 to 2003. *Ecohealth* **7**, 294–306 (2010).

2. Robinson, R. A. *et al.* Emerging infectious disease leads to rapid population declines of common british birds. *PLoS One* **5**, e12215 (2010).

3. Robinson, R. A., Morrison, C. A. & Baillie, S. R. Integrating demographic data: Towards a framework for monitoring wildlife populations at large spatial scales. *Methods Ecol. Evol.* **5**, 1361–1372 (2014).

4. Newson, S. E., Evans, K. L., Noble, D. G., Greenwood, J. J. D. & Gaston, K. J. Use of distance sampling to improve estimates of national population sizes for common and widespread breeding birds in the UK. *J. Appl. Ecol.* **45**, 1330–1338 (2008).

5. Newson, S. E., Massimino, D., Johnston, A., Baillie, S. R. & Pearce-Higgins, J. W. Should we account for detectability in population trends? *Bird Study* **60**, 384–390 (2013).

6. Baillie, S. R. The contribution of ringing to the conservation and management of bird populations: A review. *Ardea* **89**, 167–184 (2001).

7. Crick, H. Q. P., Baillie, S. R. & Leech, D. I. The UK Nest Record Scheme: its value for science and conservation. *Bird Study* **50**, 254–270 (2003).

8. Cramp, S. *Handbook of the Birds of Europe, the Middle East and North Africa. Volume VIII: Crows to Finches.* (Oxford University Press, 1994).

9. Scott, A., Christie, M. & Midmore, P. Impact of the 2001 foot-and-mouth disease outbreak in Britain: Implications for rural studies. *J. Rural Stud.* **20**, 1–14 (2004).

10. Ridout, M. S. & Besbeas, P. An empirical model for underdispersed count data. *Stat. Model.* **4**, 77–89 (2004).

11. Robinson, R. A. BirdFacts: profiles of birds occurring in Britain & Ireland. *BTO, Thetford* (2005). Available at: http://www.bto.org/birdfacts. (Accessed: 15th January 2022)

12. Kéry, M. & Schaub, M. *Bayesian Population Analysis using WinBUGS: a hierarchical perspective*. (Academic Press, Elsevier, 2012).

13. Robinson, R. A., Green, R. E., Baillie, S. R., Peach, W. J. & Thomson, D. L. Demographic mechanisms of the population decline of the song thrush *Turdus philomelos* in Britain. *J. Anim. Ecol.* **73**, 670–682 (2004).

14. Robinson, R. A., Baillie, S. R. & King, R. Population processes in European Blackbirds *Turdus merula*: A state-space approach. *J. Ornithol.* **152**, 419–433 (2012).

15. Abadi, F., Gimenez, O., Arlettaz, R. & Schaub, M. An assessment of integrated population models: bias, accuracy, and violation of the assumption of independence. *Ecology* **91**, 7–14 (2010).

16. Woodward, I. D. *et al.* *BirdTrends 2020: trends in numbers, breeding success and survival for UK breeding birds*. (2020).

17. Gill, F., Donsker, D. & Rasmussen, P. IOC World Bird List 12.1. (2022). doi:10.14344/IOC.ML.12.1
